# Associations of circulating insulin-like growth factor-I with intake of dietary proteins and other macronutrients

**DOI:** 10.1016/j.clnu.2021.04.021

**Published:** 2021-07

**Authors:** Cody Z. Watling, Rebecca K. Kelly, Tammy Y.N. Tong, Carmen Piernas, Eleanor L. Watts, Sandar Tin Tin, Anika Knuppel, Julie A. Schmidt, Ruth C. Travis, Timothy J. Key, Aurora Perez-Cornago

**Affiliations:** a Cancer Epidemiology Unit, Nuffield Department of Population Health, University of Oxford, Oxford, United Kingdom; b Nuffield Department of Primary Care Health Sciences, University of Oxford, Oxford, United Kingdom

**Keywords:** Protein, Macronutrients, Alcohol, IGF-I, Somatomedin C, Biomarker, BMI, Body mass index, HRT, hormone replacement therapy, IGF-I, insulin-like growth factor-I, IGFBP, insulin-like growth factor binding proteins, CI, confidence interval, SCFA, short chain fatty acids, SD, standard deviation

## Abstract

**Background & aims:**

Circulating insulin-like growth factor-I (IGF-I) is associated with the risk of several cancers. Dietary protein intake, particularly dairy protein, may increase circulating IGF-I; however, associations with different protein sources, other macronutrients, and fibre are inconclusive. To investigate the associations between intake of protein, macronutrients and their sources, fibre, and alcohol with serum IGF-I concentrations.

**Methods:**

A total of 11,815 participants from UK Biobank who completed ≥4 24-h dietary assessments and had serum IGF-I concentrations measured at baseline were included. Multivariable linear regression was used to assess the cross–sectional associations of macronutrient and fibre intake with circulating IGF-I concentrations.

**Results:**

Circulating IGF-I concentrations were positively associated with intake of total protein (per 2.5% higher energy intake: 0.56 nmol/L (95% confidence interval: 0.47, 0.66)), milk protein: 1.20 nmol/L (0.90, 1.51), and yogurt protein: 1.33 nmol/L (0.79, 1.86), but not with cheese protein: −0.07 nmol/L (−0.40, 0.25). IGF-I concentrations were also positively associated with intake of fibre (per 5 g/day higher intake: 0.46 nmol/L (0.35, 0.57)) and starch from wholegrains (Q5 vs. Q1: 1.08 nmol/L (0.77, 1.39)), and inversely associated with alcohol consumption (>40 g/day vs <1 g/day: −1.36 nmol/L (−1.00, −1.71)).

**Conclusions:**

These results show differing associations with IGF-I concentrations depending on the source of dairy protein, with positive associations with milk and yogurt protein intake but no association with cheese protein. The positive association of fibre and starch from wholegrains with IGF-I warrants further investigation.

## 1 Introduction

Insulin-like growth factor I (IGF-I) is a peptide-hormone involved in regulating cell growth, differentiation, and proliferation [1]. Primarily produced in the liver under stimulation by growth hormone, IGF-I mainly acts by binding to the IGF-I receptor, found on the cell membranes in most tissues in the body [1]. In prospective cohort studies and Mendelian randomization studies, higher circulating IGF-I concentrations have been associated with higher risks of breast, colorectal, and prostate cancer [[2], [3], [4], [5], [6]]. In contrast, evidence from Mendelian randomization studies suggest that higher IGF-I concentrations are associated with greater bone mineral density and a lower risk of fractures [7]. Differing IGF-I concentrations arise from a combination of both non-modifiable and modifiable factors. Associations of modifiable factors with circulating IGF-I concentrations are not well understood and identifying modifiable factors for IGF-I concentrations may be important for cancer prevention.

Dietary intake has been suggested to influence circulating IGF-I [[8], [9], [10], [11]]. Evidence from small observational studies and randomized controlled trials have suggested that higher intake of protein [9,10,12,13] and dairy products [10], possibly due to their protein content [8], may increase IGF-I concentrations. However, the associations of circulating IGF-I concentrations with different sources of protein and other nutrients have not been well characterised in previous studies and have been limited by small sample sizes with the inability to look at sources of protein in detail [[8], [9], [10],[13], [14], [15]].

The UK Biobank cohort study measured IGF-I concentrations in nearly all participants as well as collected detailed dietary information on a subsample of participants. Using this resource, we conducted an observational analysis to assess the associations of intake of protein from differing sources and other macronutrients with circulating IGF-I concentrations.

## 2 Materials and methods

### 2.1 Study design

The UK Biobank is a large British cohort established to assess the association of a wide range of exposures with disease risk [16]. Individuals were identified using the National Health Service patient registers and 9.2 million people were invited to participate by attending a visit at one of the 22 assessment centres. In total 503,317 individuals aged 37–73 years consented to enrol in the UK Biobank from 2006 to 2010 [16]. At recruitment, participants provided informed consent and detailed information about lifestyle, sociodemographic, and reproductive factors via a touchscreen questionnaire. Anthropometric measurements were conducted using standardized procedures [17], and blood samples were taken [18]. Ethical approval was obtained through the North West Multi-Centre Research Ethics Committee (reference number 16/NW/0274). A full description of the study assessment, protocol, and ethical approval can be found on the UK Biobank website [19].

### 2.2 Dietary assessment: Oxford WebQ – 24-h dietary assessment

For a subsample of the UK Biobank, the Oxford WebQ, a validated web-based 24-h dietary assessment, from which nutrient intakes can be estimated, was completed multiple times [20].

The Oxford WebQ 24-h dietary assessment questionnaire asks participants to recall the frequency of consumption of 206 types of foods and 32 types of drinks they consumed during the previous 24 h [21]. Validation of the Oxford WebQ has been conducted in a sample of 160 men and women with recovery biomarker measurements for protein, potassium, and sugar, and accelerometer for energy [20]. The correlation between protein intake estimated by four 24-h dietary assessments and recovery biomarker for protein intake (nitrogen excretion) was 0.52 (95% confidence interval (CI): 0.37–0.67) [20].

For the last 70,747 participants who were recruited between April 2009 and September 2010, the Oxford WebQ was completed at their recruitment visit. Moreover, every 3–4 months for a total of four times between February 2011 and April 2012, a link to the 24-h dietary assessment was emailed to all participants who had provided a valid email address at recruitment (Supplementary Figure S1). During the follow-up period, the response rate in each follow-up 24-h dietary assessment varied between 26% and 33% of participants with valid emails responding. In this study, participants were eligible to be included if they completed in a minimum of four (maximum five) 24-h dietary assessments in total, with one being completed at the recruitment visit in order to have one dietary assessment at the same time that the blood sample was taken.

Macronutrients and their sources, fibre, and energy intake were estimated in each completed 24-h dietary assessment from food and beverages [22,23]. Percentage of energy from specific macronutrients were calculated for each 24-h dietary assessment and intake from a minimum of four (maximum five) 24-h dietary assessments were averaged to estimate usual percentage of energy intake. Fibre intake was estimated in grams for each 24-h dietary assessment and averaged from four (maximum five) 24-h dietary assessments. For alcohol intake, participants were categorised by: <1 g/day, 1–9.9 g per day, 10–19.9 g/day, 20–39.9 g/day, and ≥40 g/day from the averaged intake of alcohol. Participants’ percentage of energy from macronutrients, fibre in grams, and energy intake were categorised into sex-specific quintiles. Full description of the calculation and categorization for macronutrients, fibre, alcohol, and energy intake can be found in the Supplementary Methods.

### 2.3 Laboratory analysis

Non-fasted blood samples were provided by 99.7% of participants at the recruitment visit and transported at 4°C to the central laboratory for cryopreservation and biochemistry measurements. Serum concentrations of IGF-I were measured using DiaSorin Ltd. LIAISON ® XL chemiluminescent immunoassay (see Supplementary Methods for further details) [18]. A total of 16,689 participants had a valid IGF-I follow-up measurement after a subsample of participants repeated the recruitment assessment and provided a second blood sample ~4 years after initial recruitment (see Supplementary Methods for further information on the repeat assessment).

### 2.4 Study population and exclusions

Our main analyses includes a total of 11,815 participants from UK Biobank who completed four (maximum five) 24-h dietary assessments and had serum IGF-I concentrations measured at baseline. In secondary analyses, we took a different sample of participants who had IGF-I re-measured at the reassessment visit (mean 4.3 years after recruitment, standard deviation (SD): 0.9 years). Participants needed to complete a minimum of four valid 24-h dietary assessments at any time (Supplementary Figure S2) to be included in this analysis. A study flowchart of exclusions for each analysis can be found in Supplementary Figure S3.

At the time of this analysis, a total of 824 participants had withdrawn their informed consent from the UK Biobank and were excluded. Participants were also excluded if they had a prevalent invasive cancer at recruitment (excluding non-melanoma skin cancer; N = 27,174), were taking medications which may alter IGF-I such as growth hormone (N = 4077; Supplementary Table S1), or did not have a value for IGF-I concentration at recruitment (N = 32,789). Participants were also excluded if they did not complete any 24-h dietary assessments (N = 251,933). As well, 24-h dietary assessments were excluded if they did not report a reliable energy intake (men: >17,575 kJ (4200 kcal) or <3347 kJ (800 kcal); women >14,644 kJ (3500 kcal) or <2092 kJ (500 kcal)) or reported to be ill or fasting on the relevant day. A total of 2439 and 592 participants were excluded because they did not have a 24-h dietary assessment with reliable energy intake or they reported to be ill or fasting, respectively. Participants were also excluded if they did not complete the 24-h dietary assessment at recruitment (N = 123,497), to ensure that at least one valid 24-h dietary assessment was completed when the participants’ blood was drawn. Finally, participants who did not complete a minimum of four valid 24-h dietary assessments were excluded to reduce random measurement error (N = 48,177). Of those who completed a 24-h dietary assessment at recruitment, 11,815 participants completed four (maximum five) valid 24-h dietary assessments and had IGF-I concentrations measured at recruitment and were included in this analysis (Supplementary Figure S2).

### 2.5 Statistical analysis

IGF-I was logarithmically transformed to obtain the geometric mean concentrations of IGF-I within each category or quintile of macronutrients, fibre, alcohol, and energy intake from linear regression models. To determine relative values, geometric means in the highest category or quintile were divided by the geometric mean in the lowest category or lowest quintile. All macronutrients were also modeled as a continuous variable in increments of 2.5% of energy intake and 5 g/day for fibre intake. A 2.5% higher energy intake was selected to account for the small variability in the macronutrients from specific sources despite the larger variability in total protein, carbohydrate, and fat, and for these analyses we used untransformed IGF-I concentrations to have a constant slope in the multivariable linear regression models.

Confounders were all selected *a priori* based on probable associations with IGF-I concentrations and dietary intake. In minimally adjusted linear regression models, adjustments were made for sex and age at recruitment (<45, 45–49, 50–54, 55–59, 60–64, ≥65 years). Multivariable linear regression models were further adjusted for region of recruitment (North-West England, North-Eastern England, Yorkshire & the Humber, West Midlands, East Midlands, South-East England, South-West England, London, Wales, and Scotland), body mass index (BMI; <20, 20–22.49, 22.5–24.9, 25–27.49, 27.5–29.9, 30–32.49, 32.5–34.9, ≥35 kg/m^2^, and unknown/missing (0.4%)), height (eight sex-specific categories increasing by 5 cm, and unknown/missing (0.5%)), physical activity (low; 0–9.99, medium; 10–49.99, high; ≥ 50 metabolic equivalent of task-hours/week, and unknown/missing (3.6%)), Townsend deprivation index (quintiles from most deprived to least deprived, and unknown/missing (0.1%)), education (completion of national exam at 16 years of age, completion of national exam at 17–18 years of age, college or university degree, or unknown/missing (17%)), smoking status (never, former, light smoker: <15 cigarettes/day, medium smoker: 15–29 cigarettes/day, heavy smoker: ≥30 cigarettes/day, or missing/unknown (0.5%)), alcohol consumption from the touchscreen questionnaire (none drinkers, <1, 1–9.99, 10–19.99, ≥20 g/day or unknown/missing (0.7%)), ethnicity (white, mixed race, Indian/Pakistani/Bangladeshi, Chinese/Asian, black/black British, other, or missing/unknown (0.5%)), diabetes status (not diabetic, diabetic, or unknown (0.4%)), energy intake (sex-specific quintiles), and women specific covariates: hormone replacement therapy (HRT) use (no, former, current, or unknown (0.3%)), oral contraceptive use (no, former, current, or unknown (0.2%)), and menopausal status (premenopausal, postmenopausal, or unknown (4.8%)). Further information on classification and categorisation of covariates can be found in the Supplementary Methods.

### 2.6 Subgroup and sensitivity analyses

We assessed whether there was heterogeneity by sex and by BMI groups (<30, and ≥30 kg/m^2^) by using a likelihood ratio test comparing the main model to a model including an interaction term between the macronutrient (modelled per 2.5% of energy intake) and sex or BMI.

In order to assess the robustness of results, sensitivity analyses with total percentages of energy from carbohydrates, fat, protein, and alcohol as well as total fibre intake, categorised into sex-specific deciles were conducted. As well, to investigate if the associations observed were independent of other nutrients, we further adjusted for the strongest observed associations, namely fibre intake (sex-specific quintiles), protein from milk (sex-specific quintiles), and protein from yogurt (sex-specific quintiles).

### 2.7 Analyses using IGF-I measurement ~4 years after recruitment (N = 2724)

Secondary analyses were conducted in participants who had IGF-I concentrations measured during the follow-up period at the reassessment visit (mean 4.3 years after recruitment). For this, participants were restricted to those who completed a minimum of four 24-h dietary assessments at any time and had a follow-up IGF-I measurement (Supplementary Figure S2 and S3). All macronutrients were added separately as 2.5% higher energy intake or 5 g/day fibre intake or modelled as quintiles in multivariable models.

All analyses were conducted using Stata version 15.1 (Stata Corp LP, College Station, TX) and “Jasper makes plots” package version 2–265 in R 3.5.2 was used to make figures. P-values were two-sided and with Bonferroni correction, p-values <0.00185 (0.05/27 exposures) were considered statistically significant. All models were visually assessed to make sure residuals were normally distributed using Q–Q plots, and not heteroscedastic using residual-versus-fitted plots. No assumptions for linear regression were deemed to be broken.

## 3 Results

The mean age of participants at recruitment was 56.7 years (SD: 7.7). Table 1 presents participant baseline characteristics by quintiles of IGF-I concentrations. Participants who had higher IGF-I concentrations were more likely to be younger, taller, have a lower BMI, report to be never smokers, less likely to be diabetic, and women were less likely to be current users of HRT.

**Table 1:** Characteristics of participants in UK Biobank WebQ 24-h dietary assessment subsample by quintiles of circulating IGF-I (N = 11,815).

	Circulating IGF-I				
	Quintile 1	Quintile 2	Quintile 3	Quintile 4	Quintile 5
Number of participants, N	2363	2363	2364	2363	2362
IGF-I concentration, nmol/L	14.6 (2.2)	18.9 (0.9)	21.7 (0.8)	24.5 (0.9)	30.1 (4.6)
IGF-I concentration at follow-up, nmol/L^a^	15.7 (3.2)	19.2 (2.9)	21.2 (3.7)	23.2 (3.2)	28.1 (5.0)
Sex - Male, N (%)	859 (36.4%)	984 (41.6%)	1042 (44.1%)	1124 (47.6%)	1120 (47.4%)
Age, years	59.4 (6.5)	58.1 (7.0)	56.6 (7.6)	55.6 (7.8)	53.5 (8.2)
Body mass index, kg/m^2^	27.3 (5.3)	26.7 (4.6)	26.2 (4.2)	26.0 (4.0)	25.9 (3.9)
Height, cm	167.5 (8.9)	169.0 (9.0)	169.8 (9.1)	170.2 (9.0)	170.9 (9.1)
Physical activity, N (%)					
Low	661 (28.0%)	615 (26.0%)	608 (25.7%)	609 (25.8%)	585 (24.8%)
Moderate	1251 (52.9%)	1262 (53.4%)	1280 (54.1%)	1273 (53.9%)	1346 (57.0%)
High	405 (17.1%)	437 (18.5%)	450 (19.0%)	452 (19.1%)	393 (16.6%)
Townsend deprivation index, N (%)					
Q1 - Most affluent	396 (16.8%)	421 (17.8%)	399 (16.9%)	431 (18.2%)	418 (17.7%)
Q5 - Most deprived	409 (17.3%)	380 (16.1%)	376 (15.9%)	366 (15.5%)	382 (16.2%)
Education, N (%)					
National Examination at age 16 years	358 (15.2%)	331 (14.0%)	300 (12.7%)	303 (12.8%)	286 (12.1%)
National Examination at age 17–18 years	145 (6.1%)	169 (7.2%)	163 (6.9%)	156 (6.6%)	152 (6.4%)
College or University degree	1707 (72.2%)	1735 (73.4%)	1778 (75.2%)	1792 (75.8%)	1829 (77.4%)
Smoking, N (%)					
Never	1290 (54.6%)	1334 (56.5%)	1380 (58.4%)	1437 (60.8%)	1471 (62.3%)
Previous	914 (38.7%)	877 (37.1%)	842 (35.6%)	798 (33.8%)	756 (32.0%)
Light smoker: <15 cigarettes/day	37 (1.6%)	42 (1.8%)	44 (1.9%)	45 (1.9%)	41 (1.7%)
Medium smoker: 15–29 cigarettes/day	47 (2.0%)	31 (1.3%)	41 (1.7%)	31 (1.3%)	30 (1.3%)
Heavy smoker: ≥30 cigarettes/day	71 (3.0%)	75 (3.2%)	55 (2.3%)	48 (2.0%)	60 (2.5%)
Alcohol intake, N (%)					
<1 g/day	526 (22.3%)	489 (20.7%)	498 (21.1%)	518 (21.9%)	520 (22.0%)
1–9.99 g/day	568 (24.0%)	559 (23.7%)	545 (23.1%)	541 (22.9%)	639 (27.5%)
10–19.99 g/day	464 (19.6%)	482 (20.4%)	480 (20.3%)	512 (21.7%)	502 (21.3%)
20–39.9 g/day	477 (20.2%)	517 (21.9%)	580 (24.5%)	535 (22.6%)	517 (21.9%)
≥ 40 g/day	328 (13.9%)	316 (13.4%)	261 (11.0%)	257 (10.9%)	184 (7.8%)
Ethnicity, N (%)					
White	2293 (97.0%)	2300 (97.3%)	2280 (96.4%)	2289 (96.9%)	2261 (95.7%)
Mixed Race	13 (0.6%)	18 (0.8%)	16 (0.7%)	16 (0.7%)	24 (1.0%)
Indian/Pakistani/Bangladeshi	18 (0.8%)	5 (0.2%)	15 (0.6%)	17 (0.7%)	15 (0.6%)
Chinese, Asian, or other Asian	10 (0.4%)	9 (0.4%)	10 (0.4%)	6 (0.3%)	10 (0.4%)
Black or Black British	10 (0.4%)	10 (0.4%)	11 (0.5%)	11 (0.5%)	27 (1.1%)
Other	11 (0.5%)	13 (0.6%)	19 (0.8%)	18 (0.8%)	16 (0.7%)
Diabetic - Yes, N (%)	147 (6.2%)	107 (4.5%)	81 (3.4%)	80 (3.4%)	79 (3.3%)
**Women-only covariates**					
Current HRT users, N (%)	187 (12.4%)	93 (6.7%)	88 (6.7%)	68 (5.5%)	58 (4.7%)
Current oral contraceptive pill users, N (%)	9 (0.6%)	16 (1.2%)	20 (1.5%)	31 (2.5%)	78 (6.3%)
Menopause status at recruitment, N (%)					
Premenopausal	114 (7.6%)	185 (13.4%)	225 (17.0%)	322 (26.0%)	479 (38.6%)
Postmenopausal	1297 (86.2%)	1083 (78.5%)	959 (72.5%)	789 (63.6%)	640 (51.5%)

Values are mean (SD) unless otherwise indicated, percentages include unknown category for missing data.

Percentages calculated including missing values and therefore may not add up to 100%.

Abbreviations: HRT, hormone replacement therapy; IGF-I, insulin-like growth factor-I; N, Number of participants; Q, quintile; SD, standard deviation.

a Participants include those who had a follow-up blood sample provided and IGF-I concentrations measured (N = 2724).

**Fig. 1:**
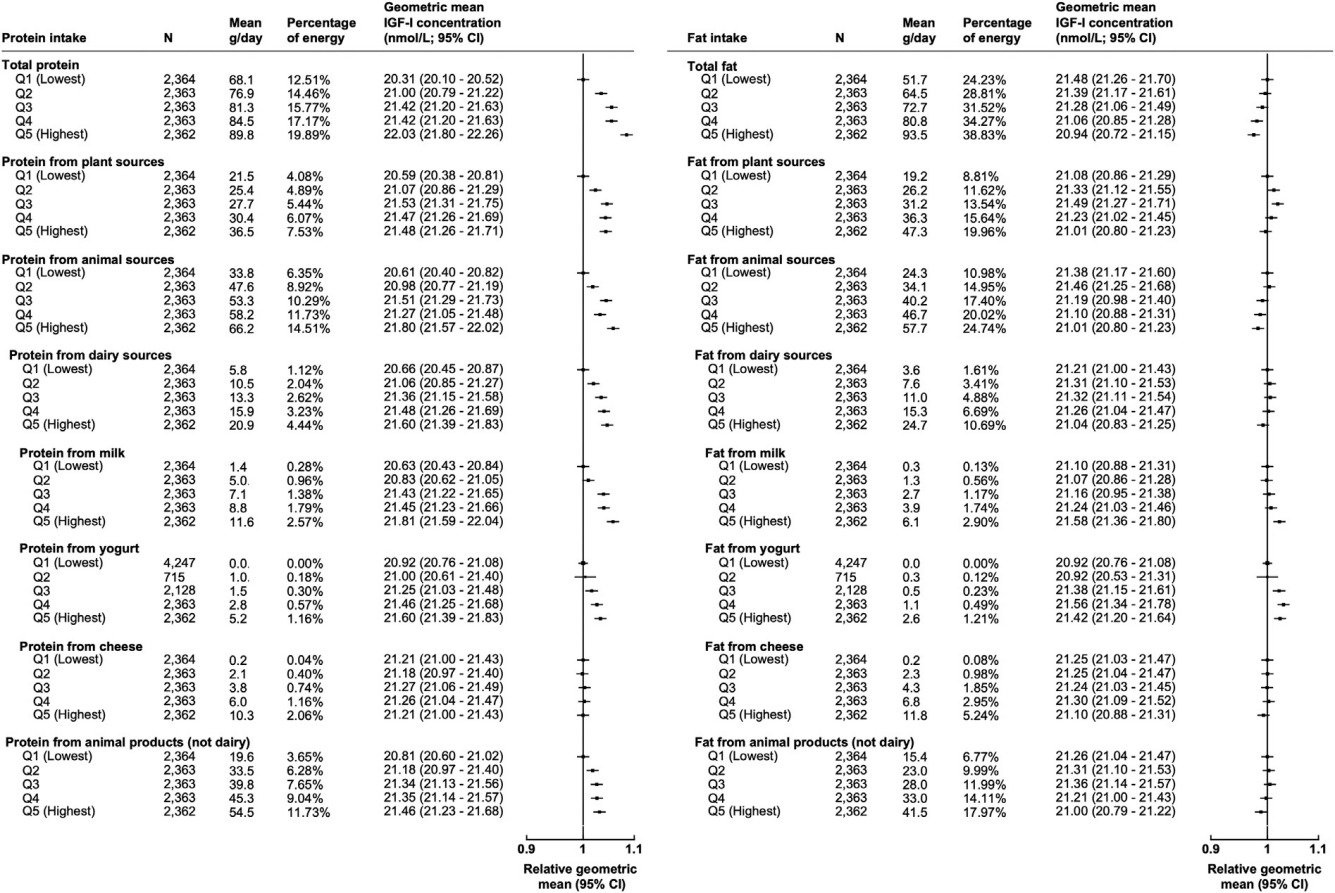
**Percentage of energy intake from proteins and fats separated by quintiles in association with geometric mean concentrations of IGF-I (N**=**11,815).** All models are adjusted for sex, age at recruitment, region of recruitment, body mass index, height, physical activity, Townsend deprivation index, education, smoking, alcohol consumption, ethnicity, diabetes status, energy intake, and women specific covariates: hormone replacement therapy use, oral contraceptive use, and menopausal status. Percentage of energy from protein and fat sources calculated from a minimum of four averaged 24-h web-based dietary assessments with one completed at recruitment. Grams calculated as mean per day within each quintile. Percentage of energy calculated by mean percentage of energy per day in each quintile. Abbreviations: CI, confidence intervals; g, grams; IGF-I, insulin-like growth factor-I; N, number of participants; Q, quintile.

**Fig. 2:**
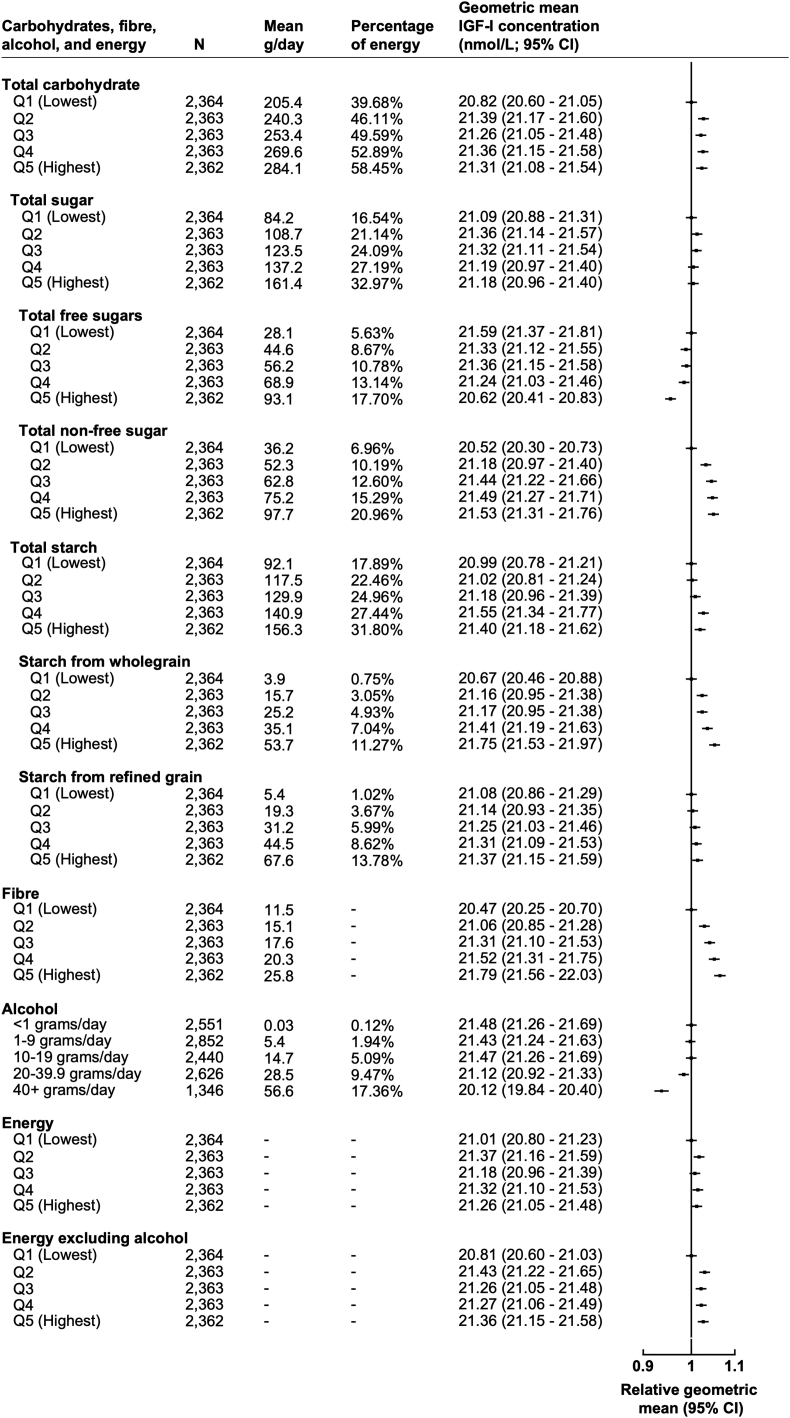
**Percentage of energy from carbohydrates sources, fibre, alcohol, and energy intake in association with geometric mean concentrations of IGF-I (N**=**11,815).** All models are adjusted for sex, age at recruitment, region of recruitment, body mass index, height, physical activity, Townsend deprivation index, education, smoking, alcohol consumption (except when alcohol was the exposure), ethnicity, diabetes status, energy intake (except when energy intake was the exposure), and women specific covariates: hormone replacement therapy use, oral contraceptive use, and menopausal status. Percentage of energy from carbohydrate sources, fibre quintiles, alcohol categories, and energy intake quintiles calculated from a minimum of four averaged 24-h web-based dietary assessments with one completed at recruitment. Grams calculated as mean per day within each quintile and category. Percentage of energy calculated by mean percentage of energy per day in each quintile. Abbreviations: CI, confidence intervals; g, grams; IGF-I, insulin-like growth factor-I; N, number of participants; Q, quintile.

**Fig. 3:**
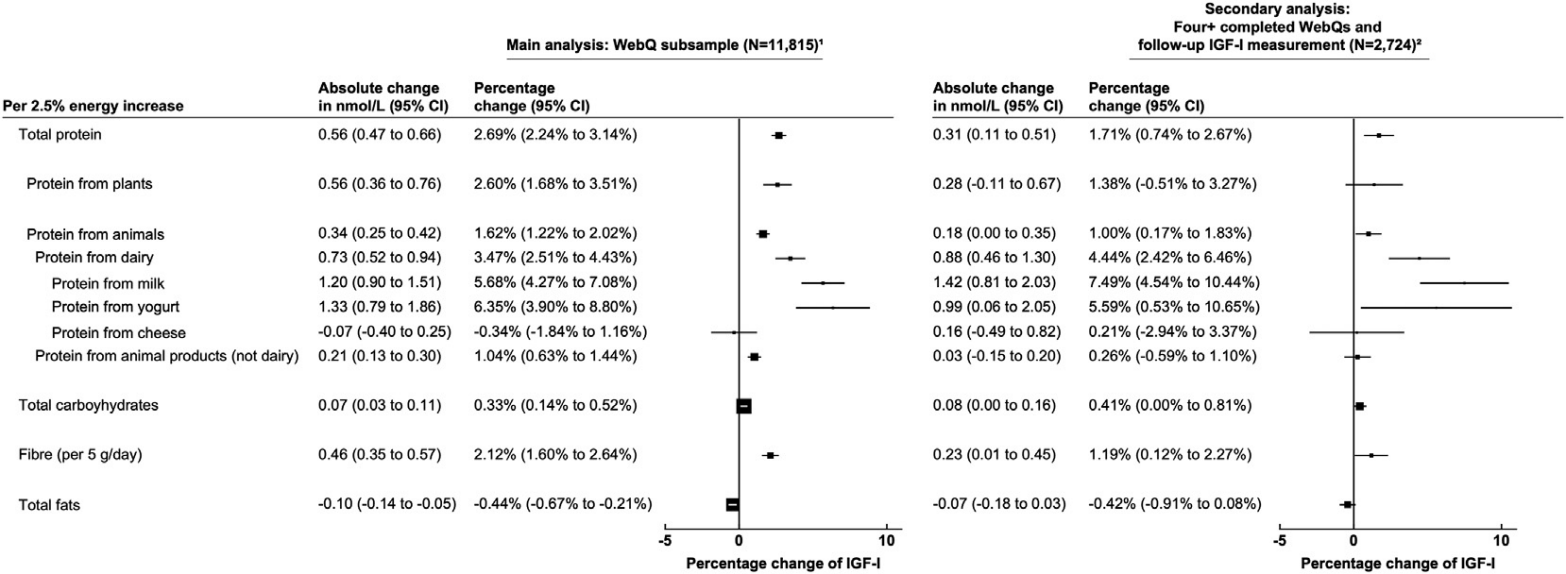
**Associations of IGF-Iwithincremental intake of energy from macronutrients and fibre (N**=**11,815) and restricting to participants with an IGF-I measurement ~4 years after recruitment (N**=**2724).** All models are adjusted for sex, age at recruitment, region of recruitment, body mass index, height, physical activity, Townsend deprivation index, education, smoking, alcohol consumption, ethnicity, diabetes status, energy intake, and women specific covariates: hormone replacement therapy use, oral contraceptive use, and menopausal status. ^1^ Macronutrients calculated from a minimum of four (maximum of five) averaged 24-h web-based diet assessments with one completed at recruitment. ^2^ Analysis assessing macronutrients and fibre intake calculated for participants with a follow-up IGF-I measurement. Macronutrients and fibre intake were calculated by using a minimum of four (maximum of five) averaged 24-h web-based dietary assessments completed at any time. IGF-I was measured after all completed WebQs (mean follow-up time from last WebQ to follow-up blood measurement: 0.64 years) and ~4 years after recruitment. Macronutrients are modelled as a 2.5% energy increase whereas fibre is modelled as a 5 g per day increase. Abbreviations: CI, confidence intervals; g/day, grams per day; IGF-I, insulin-like growth factor-I.

The main results from the multivariable adjusted models are described below if the association between macronutrient, fibre, alcohol, or energy intake was associated with a ≥5% difference in circulating IGF-I concentrations (Fig. 1, Fig. 2, Fig. 3; Supplementary Tables S2 and S3). The minimally adjusted results for the intake of macronutrients, energy, alcohol, and fibre in relation to IGF-I concentrations are shown in Supplementary Figures S4-S6.

Results for macronutrient intake by quintiles are shown in Fig. 1, Fig. 2 and by lowest versus highest quintile in Supplementary Table S2. Comparing participants in the highest quintile with participants in the lowest quintile of intake for total protein, protein from animal sources, and protein from milk, IGF-I concentrations were 1.72 nmol/L (95% CI: 1.40, 2.03), 1.19 nmol/L (95% CI: 0.88, 1.50), and 1.18 nmol/L (95% CI: 0.87, 1.49) higher, respectively (Fig. 1 & Supplementary Table S2). Fat intake was not associated with notable differences in circulating IGF-I concentrations.

Participants in the highest quintile of fibre intake had 1.32 nmol/L (95% CI: 1.00, 1.65) higher IGF-I concentrations in comparison to the lowest quintile (Fig. 2). Circulating IGF-I concentrations were 1.08 nmol/L (95% CI: 0.77, 1.39) higher in the highest quintile of percentage of energy from starch from wholegrains intake in comparison to the lowest quintile. Circulating IGF-I concentrations were significantly lower for participants reporting consuming ≥40 g of alcohol a day in comparison to those who reported drinking <1 g of alcohol per day (−1.36 nmol/L, 95% CI: −1.00, −1.71; Fig. 2).

A 2.5% higher intake of energy from total protein was associated with higher IGF-I concentrations by 0.56 nmol/L (95% CI: 0.47, 0.66; Fig. 3). When looking at specific sources of protein, a 2.5% higher intake in energy from protein from dairy was associated with 0.73 nmol/L (95% CI: 0.52, 0.94) higher IGF-I concentrations. When we looked at dairy sources in more detail, intake of protein from milk and protein from yogurt were associated with higher IGF-I concentrations of 1.20 nmol/L (95% CI: 0.90, 1.51), and 1.33 nmol/L (95% CI: 0.79, 1.86), respectively; however, there was no association between protein from cheese and IGF-I concentrations (−0.07 nmol/L; 95% CI: −0.40, 0.25; Fig. 3 and Supplementary Table S4).

### 3.1 Subgroup and sensitivity analyses

No evidence of heterogeneity was observed between sex and BMI subgroups for macronutrients and fibre intake (Supplementary Figures S7 and S8).

The patterns of associations remained the same when percentages of energy from macronutrients and fibre were modelled as deciles (Supplementary Figure S9). In the multivariable models controlling for fibre intake, the associations of intake of protein and fat from various sources with circulating IGF-I concentrations remained largely the same (Supplementary Figures S10 & S11). When intake of milk protein and yogurt protein were added to the multivariable models, the association between starch from wholegrains and IGF-I concentrations became attenuated, whereas the association with fibre intake was not materially affected (Supplementary Figure S12).

### 3.2 Analyses using IGF-I measurement ~4 years after recruitment (N = 2724)

In secondary analyses using participants with IGF-I measured ~4 years after recruitment who had completed a minimum of four 24-h dietary assessments, the results were largely similar in comparison to using the recruitment IGF-I measurements; however, the association of IGF-I with protein from milk intake was stronger (per 2.5% energy increase: 1.42 nmol/L (95% CI: 0.81, 2.03); Fig. 3 and Supplementary Table S4). The associations with protein from yogurt, and fibre intake, became slightly attenuated (per 2.5% energy increase: 0.99 nmol/L (95% CI: 0.06, 2.05) and per 5 g/day increase: 0.23 nmol/L (95% CI: 0.01, 0.45), respectively; Fig. 3 and Supplementary Table S4).

## 4 Discussion

In this observational analysis in UK Biobank, we found that the association of circulating IGF-I concentrations with protein from dairy products differed by sources of dairy, with protein from milk and yogurt being positively associated with IGF-I concentrations, whereas protein from cheese was not associated with circulating IGF-I concentrations. We also found positive associations between total protein intake, fibre, and starch from wholegrains and circulating IGF-I concentrations, and an inverse association with alcohol consumption. Our findings showing different associations with IGF-I depending on the source of dairy protein are potentially important, and together with the positive associations of dietary fibre and starch from wholegrains intake with IGF-I warrant further investigation.

### 4.1 Protein

Similar to previous cross-sectional analyses [[8], [9], [10],^24^], higher total protein intake was associated with higher circulating concentrations of IGF-I. This association has also been shown in some randomized controlled trials of dairy-based protein supplements, which used whey and/or milk protein isolate [15,25], and a trial assessing isocaloric protein restriction where reduction of protein intake resulted in lower IGF-I concentrations [26]. Circulating IGF-I concentrations have also been shown to be lower in individuals who are vegan [27,28]; a diet which contains no dairy and usually less protein than average [29].

### 4.2 Protein from dairy

Although protein from all dairy products combined was associated with higher IGF-I concentrations, we found that intake of protein from milk and yogurt was strongly associated with circulating IGF-I, whereas there was no evidence of an association with protein from cheese, suggesting a potential difference between dairy sources. In analyses using the IGF-I measurement ~4 years after recruitment, the association of protein from milk with IGF-I concentrations became stronger whereas protein from yogurt became slightly attenuated, although was still positively associated with IGF-I. Previous studies have also reported a positive association between total dairy protein intake and IGF-I [8,24], as well as a possible null association between cheese intake and IGF-I concentrations [10,30,31], although results are inconsistent [32], and to our knowledge, evidence on protein from different dairy products has not been previously explored. As a possible reason for this difference by dairy subtype, whey protein is found in milk and yogurt, but is removed in most cheese production. Whey contains relatively more branched chained amino acids (leucine, isoleucine, and valine) and is more quickly absorbed into the bloodstream in comparison with casein, the protein found in cheese [33], although it is unclear whether whey protein increases IGF-I more than casein protein [34]. The supply of essential amino acids, such as tryptophan, may be particularly important in the up-regulation of IGF-I genes [35,36] and signalling pathways in the liver necessary for IGF-I synthesis [37]. Other components in dairy products such as calcium may also affect IGF-I concentrations [8,9,24,32], but evidence is unclear [38].

### 4.3 Fibre, starch from wholegrains, and alcohol

We also found that higher fibre and wholegrain intakes were associated with higher circulating concentrations of IGF-I, even after controlling for protein from milk and yogurt. The finding for fibre is consistent with two cross-sectional studies of 4731 individuals and 1037 women [8,10], although other smaller cross-sectional studies have not found evidence of an association [39,40]. Wholegrains are rich in fibre, and it is possible that the association with IGF-I may be due to their fibre content. Fibre might influence IGF-I concentrations via the gut microbiota; anaerobic intestinal microbiota such as *Lactobacillus* and *Bifidobacterium* ferment fibre in the colon to produce short-chain fatty acids (SCFA) [41], and some research using mice models suggests that SCFA supplementation may increase IGF-I concentrations [41,42]. Further research is required to determine whether fibre may increase IGF-I concentrations in humans through SCFA or other mechanisms.

In line with previous studies [40,43], participants consuming the highest amount of alcohol (≥40 g/day) had lower concentrations of IGF-I. This may be due to ethanol toxicity, which impairs the hepatic synthesis of IGF-I [44].

### 4.4 Strengths and limitations

This study has several strengths. This is currently the largest analysis to be conducted assessing macronutrient intake in relation to circulating IGF-I concentrations, therefore we have high statistical power to detect associations. Multiple 24-h dietary assessments were used to estimate the usual macronutrient intake, which reduces random measurement error in the estimates. We were also able to test the robustness of our results using the follow-up measurement of IGF-I, which was taken an average of 0.64 years after the last 24-h dietary assessment was completed.

There are some limitations to consider. Only a subsample of the participants in the UK Biobank completed the 24-h dietary assessment several times, although this subsample is larger than any previous study assessing these associations [8]. Although a minimum of four 24-h dietary assessments were completed by this subsample, dietary intakes estimated using these assessments are still subject to measurement error and inaccuracies in self-report, which may bias these results. As well, the nature of this analysis did not allow us to consider temporality as most 24-h dietary assessments were completed several months after participants’ blood was drawn. We did consider this in the secondary analyses restricted to participants with IGF-I measured ~4 years after recruitment, and the results were largely similar and, in some cases, stronger. These analyses also did not consider other related components of the IGF signalling pathway, such as IGF-II and the insulin-like growth factor binding proteins (IGFBP), as they were not measured in this cohort. Most notably, IGFBP-3, which binds the majority of IGF-I in circulation [45], may be associated with dietary intake [9,10] and thus, together with possible dietary impacts on IGFBP-1 and IGFBP-2 [27], the bioavailability of IGF-I may be different than the observed associations, which may be important for disease risk. The UK Biobank participants are predominantly white and generally healthier than the overall population [46]. As well, participants in this subsample had to complete a minimum four 24-h dietary assessments, which introduces potential selection bias as these individuals are likely to be healthier than those not responding several times [47]; as a result, the estimates may not be generalizable to a wider population. Finally, the associations may be subject to unmeasured and residual confounding, and causality cannot be inferred.

## 5 Conclusion

In conclusion, our results show differing associations with IGF-I depending on the source of dairy protein, with intake of protein from milk and yogurt being associated with IGF-I concentrations, whereas intake of protein from cheese was not associated with circulating IGF-I concentrations. We also found positive associations between total protein intake, fibre, and starch from wholegrains and circulating IGF-I concentrations, and an inverse association with alcohol consumption. The positive association of fibre and starch from wholegrains with IGF-I is not well characterised and warrants further investigation. Further research assessing individual amino acids, as well as using methods that may be less susceptible to residual confounding, including large randomized controlled trials and Mendelian randomization studies, are needed to enhance understanding of how dietary components may modulate IGF-I concentrations.

## Ethics approval and consent to participate

The UK Biobank study obtained ethical approval through the North West Multi-Centre Research Ethics Committee (reference number 16/NW/0274). All participants provided informed consent to participate at recruitment.

## Funding information

This work is supported by the Nuffield Department of Population Health Doctor of Philosophy student scholarship and by Cancer Research UK (C8221/A29017). RKK is supported by the Clarendon Scholarship from the University of Oxford. TYNT is supported by the UK Medical Research Council (MR/M012190/1). CP is supported by the Oxford and Thames Valley NIHR Applied Research Centre. ELW is supported by the Nuffield Department of Population Health Early Career Research Fellowship. STT is supported by the Girdlers’ New Zealand Health Research Council Fellowship (19/031). AK is supported by the Wellcome Trust, Our Planet Our Health (Livestock, Environment and People – LEAP; 205212/Z/16/Z). APC is supported by a Cancer Research UK Population Research Fellowship (C60192/A28516) and by the World Cancer Research Fund (WCRF UK), as part of the Word Cancer Research Fund International grant programme (2019/1953).

## Author contributions

APC and TJK conceived the research. CZW analysed the data and produced the tables and figures. CZW and APC were responsible for drafting the manuscript. All authors provided advice on the study design, analysis, and interpretation of the results. All of the authors read and approved the final manuscript.

## Conflict of interest

All authors report no conflicts of interests to disclose.
