# Supplementary material for: Associations of circulating insulin-like growth factor-I with intake of dietary proteins and other macronutrients
Source: Clin Nutr. 2021 Jul;40(7):4685–93. doi: 10.1016/j.clnu.2021.04.021 (PMC8345002; doi:10.1016/j.clnu.2021.04.021)
Supplement: Multimedia component 1 [file mmc1.pdf]

Associations of circulating insulin-like growth factor-I with intake of dietary proteins and other macronutrients

Cody Z. Watling[1], Rebecca K. Kelly[1], Tammy Y. N. Tong[1], Carmen Piernas[2], Eleanor L. Watts[1], Sandar Tin Tin[1], Anika Knuppel[1], Julie A. Schmidt[1], Ruth C. Travis[1], Timothy J. Key[1], Aurora Perez-Cornago[1]

[1] Cancer Epidemiology Unit, Nuffield Department of Population Health, University of Oxford, Oxford, United Kingdom

[2] Nuffield Department of Primary Care Health Sciences, University of Oxford, Oxford, United Kingdom

Supplementary Materials and Methods

Supplemental Methods **4**

Web-based 24-hour dietary assessment (WebQ) subsample 4

Laboratory analyses 9

Medications 10

Covariates 10

Supplementary Tables **17**

Supplementary Table S1. List of medications and UK Biobank codes from baseline used to exclude participants taking medication which may modify insulin-like growth factor-I concentrations 17

Supplementary Table S2. Geometric mean difference and percentage change of IGF-I comparing the highest quintile to the lowest quintile of nutrients using the baseline IGF-I measurement and in secondary analyses using the follow-up IGF-I measurement ~4 years after recruitment 19

Supplementary Table S3. Macronutrients and fibre from the 24-hour dietary assessment subsample ranked by largest baseline IGF-I concentration percentage difference between highest and lowest category and by 2.5% energy increase for macronutrients and 5 g/day increase for fibre 21

Supplementary Table S4. Macronutrient per 2.5% of energy intake in association with absolute difference and percentage change of IGF-I for the WebQ 24-hour dietary assessment subsample (N=11,815) and in secondary analysis for participants who completed four or more WebQs and had IGF-I measured ~4 years after recruitment (N=2,724) 22

Supplementary Figures **24**

Supplementary Figure S1. Dietary assessment for participants in the UK Biobank over time 24

Supplementary Figure S2. Dietary assessment for participants with follow-up blood sample in the UK Biobank over time 25

Supplementary Figure S3. Flow chart of exclusion criteria for WebQ 24-hour dietary assessment subsample, and secondary analysis restricting to participants who had a follow-up IGF-I measurements 26

Supplementary Figure S4. Minimally adjusted models for percentage of energy intake from proteins and fats separated by quintiles in association with geometric mean concentrations of IGF-I in the WebQ 24-hour dietary assessment subsample (N=11,815) 27

Supplementary Figure S5. Minimally adjusted models for percentage of energy from carbohydrates sources, fibre, alcohol, and energy intake in association with geometric mean concentrations of IGF-I in the WebQ 24-hour dietary assessment subsample (N=11,815) 29

Supplementary Figure S6. Minimally adjusted models for 2.5% incremental intake of energy from macronutrients and 5 grams per day fibre intake in association with the absolute and percentage change concentration of IGF-I 31

Supplementary Figure S7. Multivariable adjusted model for nutrient intake per 2.5% increase of energy from carbohydrates, fats, and proteins and 5 grams per day increase in fibre by sex in association with IGF-I 32

Supplementary Figure S8. Multivariable-adjusted model for nutrient intake per 2.5% increase of energy from carbohydrates, fats, and proteins and 5 grams per day increase in fibre by body mass index (<30 and ≥30 kg/m^2) in association with IGF-I 33

Supplementary Figure S9. Multivariable-adjusted model for percentage of energy in deciles from several macronutrients and fibre from the WebQ 24-hour dietary assessment subsample in association with the geometric mean concentration of IGF-I (N=11,815)..35

Supplementary Figure S10. Multivariable adjusted model for percentage of energy from protein adjusting for fibre intake in association with geometric mean concentration of IGF-I.....................37

Supplementary Figure S11. Multivariable adjusted model for percentage of energy from fats adjusting for milk and yogurt protein and fibre intake in association with geometric mean concentration of IGF-I...38

Supplementary Figure S12. Multivariable adjusted model for percentage of energy from carbohydrates, and alcohol, and energy further adjusting for milk and yogurt protein intake and fibre intake in association with geometric mean concentration of IGF-I..41

Supplemental Methods

Web-based 24-hour dietary assessment (WebQ) subsample

To be included in 24-hour dietary assessment (WebQ) subsample of the UK Biobank for this analysis, participants needed to complete a minimum of four 24-hour dietary assessments (maximum five) with one being completed at the recruitment visit when participants' blood samples were drawn. A minimum of four 24-hour dietary assessments were chosen to reduce random measurement error as a result of the day-to-day variation in dietary intake. Intakes of the four to five completed 24-hour dietary assessments were then averaged for all foods and nutrients in order to have usual intake.

For the WebQ 24-hour dietary assessment, participants' responses for each food were converted into respective nutrient profiles. To estimate intake, the serving size in grams was multiplied by the frequency reported in the 24-hour dietary assessment[1, 2].

<u>Macronutrient calculations</u>

The following intake of macronutrients were determined from all foods consumed in each WebQ 24-hour dietary assessment: carbohydrates, total sugars, free sugars, starch, fat, fat from vegetables, fat from animal products, protein, protein from plants, protein from animal products[2].

Grams of carbohydrates and proteins were multiplied by 16.7 to obtain the kilojoule (kJ) of energy from the specific macronutrient. Fats were multiplied by 37.7 to determine the kJ of energy from fat. Once this was determined from all WebQ 24-hour dietary assessments, each macronutrient was divided by the total amount of energy intake for the specific WebQ for

each participant to determine the percentage of energy from the macronutrient for each individual WebQ completed.

Starch from wholegrain

Starch from wholegrain was calculated from the grams of starch from wholegrain bread, wholegrain pasta or brown rice, bran cereal, biscuit cereal (Weetabix), oats (with or without sugar) and muesli. The total amount of starch in grams was summed for each WebQ 24-hour dietary assessment and multiplied by 16.7 to determine the energy from starch from wholegrains for that specific WebQ. Energy from starch from wholegrains for each WebQ were divided by the total energy intake for each specific WebQ.

Starch from refined grain

Starch from refined grain was calculated from the grams of starch from white bread, other breads, white pasta or rice, and other cereals. The total amount of starch in grams was summed for each WebQ and multiplied by 16.7 to determine the energy from starch from refined grains for each specific WebQ. Energy from starch from wholegrains for each WebQ were divided by the total energy intake for the specific WebQ.

Non-free sugars

Non-free sugars were calculated by subtracting free sugars from total sugars calculated for each WebQ and multiplied by 16.7 to obtain the kJ of energy from non-free sugars.

Protein and fat from dairy sources

Protein from dairy and fat from dairy were calculated from the grams of protein and fat from all types of milk, cream, butter, yogurt, and cheese. The total amount of dairy protein in

grams was summed for each WebQ 24-hour dietary assessment and multiplied by 16.7 to determine the energy from dairy protein for that specific WebQ. The total amount of dairy fat in grams was summed for each WebQ and multiplied by 37.7 to determine the energy from dairy fat for that specific WebQ. Energy from dairy protein and dairy fat for each WebQ were divided by the total energy intake for the specific WebQ.

Protein and fat from animal sources (not dairy)

Protein and fat from animal sources other than dairy were calculated for each participant by subtracting the total amount of energy from animal protein and animal fat from the calculated dairy protein and dairy fat for each WebQ, respectively. The total amount of animal protein not from dairy was summed for each WebQ and multiplied by 16.7 to determine the energy from animal protein not from dairy for that specific WebQ. The total amount of animal fat not from dairy was summed for each WebQ and multiplied by 37.7 to determine the energy from animal fat not from dairy for that specific WebQ. The protein and fat from animal sources not from dairy were divided by total energy intake for each WebQ.

Protein and fat from milk

Protein from milk and fat from milk were calculated from the reported intake of whole milk, semi-skimmed milk, and skimmed milk in grams in the WebQ. The protein and fat from milk were divided by each total energy WebQ. The total amount of milk protein was summed for each WebQ and multiplied by 16.7 to determine the energy from milk protein for that specific WebQ. The total amount of milk fat was summed for each WebQ and multiplied by 37.7 to determine the energy from milk fat for that each WebQ.

Protein and fat from yogurt

Protein from yogurt and fat from yogurt were calculated from the reported intake of full fat yogurt and non-fat yogurt in grams in the WebQ. The protein and fat from yogurt were divided by each total energy WebQ. The total amount of yogurt protein was summed for each WebQ and multiplied by 16.7 to determine the energy from yogurt protein for each specific WebQ. The total amount of yogurt fat was summed for each WebQ and multiplied by 37.7 to determine the energy from yogurt fat for that specific WebQ. The protein and fat from yogurt were divided by total energy intake for each WebQ.

Protein and fat from cheese

Protein from cheese and fat from cheese were calculated from the reported intake of low-fat cheese and normal cheese in grams in the WebQ. The protein and fat from cheese were divided by each total energy WebQ. The total amount of cheese protein was summed for each WebQ and multiplied by 16.7 to determine the energy from cheese protein for that specific WebQ. The total amount of cheese fat was summed for each WebQ and multiplied by 37.7 to determine the energy from cheese fat for each WebQ. The protein and fat from cheese were divided by total energy intake for each WebQ.

<u>Energy, Fibre, and Alcohol intake</u>

Total energy and energy not from alcohol

Total energy intake was calculated for each food/beverage item including alcohol intake in the WebQ in kJ and was summed to determine total energy intake for each WebQ. Total energy intake was averaged across all completed WebQs for each participant and were split into sex-specific quintiles based on the kJ each participant consumed on average. Similarly,

for total energy not from alcohol, energy was calculated for each food item excluding alcohol intake.

Fibre

Fibre intake was calculated for each food item in the WebQ in grams and was summed across as total fibre intake for each WebQ. Total fibre intake was averaged across all completed WebQs for each participant and were put into sex-specific quintiles based on the average grams of fibre each participant consumed.

Alcohol

Alcohol intake was determined from the grams of alcohol in a standard drink asked in the WebQ. Grams of alcohol were calculated from wine, spirits, and beer reported in the WebQ. Alcohol consumption was then categorised as <1 g/day, 1-9.99 g/day, 10-19.99 g/day, 20-30.99 g/day and ≥40 g/day.

<u>Average gram intake per day</u>

The average grams consumed were calculated within each category or quintile of intake of macronutrients, fibre, and alcohol from the 24-hour dietary assessments.

<u>Average percentage of energy intake per day</u>

For macronutrients, the average percentage of energy consumed per day was calculated within each quintile for all of the macronutrients.

Laboratory analyses

Blood was drawn from non-fasting participants into serum separator vacutainer tubes with clot activators, which were centrifuged and stored at 4°C before being transported to the central laboratory for cryopreservation and biochemistry measurements. Full description of storage and handling can be found on the UK Biobank website (https://biobank.ctsu.ox.ac.uk/crystal/crystal/docs/Bloodsample.pdf) as well as full details of the blood collection for the UK Biobank have been described here[3].

Serum concentrations of IGF-I were measured using a chemiluminescent immunoassay with LIAISON® XL by DiaSorin Ltd. Details about assay methods and quality control procedures for serum blood measurements are available online (https://biobank.ndph.ox.ac.uk/showcase/showcase/docs/serum_biochemistry.pdf). The coefficient of variation for circulating IGF-I concentrations at baseline was 0.265.

Repeat assessment

Participants who lived within a 35 km radius were invited to attend a repeat baseline assessment at the UK Biobank Centre in Stockport between August 2012 to June 2013. Repeat assessments were completed in 20,343 men and women with an overall response rate of 21%. Further information on the UK Biobank repeat visit can be found on the UK Biobank website (https://biobank.ctsu.ox.ac.uk/~bbdatan/Repeat_assessment_doc_v1.0.pdf).

In total 16,689 participants had IGF-I remeasured at the repeat assessment with 15,419 participants having both IGF-I measurements. Pearson correlations between the baseline IGF-I measurement and second measurement for the same individuals were r = 0.76 for all, r=0.77 for men, and r=0.74 for women.

Medications

Participants who reported to be taking medications deemed to potentially alter IGF-I concentrations at recruitment were excluded from this analysis. Supplementary Table 1 presents a list of medications that met criteria for exclusion for this analysis.

Covariates

Region

Region of participants were grouped based on the recruitment centre they attended. A total of 10 regions were used corresponding approximately to the areas covered by the assessment centre: London (assessment centres: St Bartholomew's Hospital, Hounslow, Croydon) Wales (assessment centres: Swansea, Wrexham, Cardiff), North-West England (assessment centres: Stockport, Manchester, Liverpool, Bury), North-East England (assessment centres: Newcastle, Middlesbrough), Yorkshire (assessment centres: Leeds, Sheffield), West Midlands (assessment centres: Stoke, Birmingham) East Midlands (assessment centre: Nottingham), South-East England (assessment centres: Oxford, Reading), South-West England (assessment centre: Bristol), Scotland (assessment centres: Glasgow, Edinburgh)).

Height

Participants were grouped into eight sex-specific categories for height. For women categories were: <150, 150-154.9, 155-159.9, 160-164.9, 165-169.9, 170-174.9, 175-179.9, ≥180 cm. For men categories were: <160, 160-164.9, 165-169.9, 170-174.9, 175-179.9, 180-184.9, 185-189.9, ≥190 cm. From this, men and women were combined into one height variable with categories going from 1 to 8, and a missing category for participants with missing data.

Body Mass Index (BMI)

Both height and weight were measured at the baseline visit and were used to determine participant's BMI. BMI was calculated by taking the participants measured weight in kilograms and dividing it by the participants squared standing height in metres. Individuals with missing data were coded into a missing category.

Alcohol intake form touchscreen questionnaire (covariate)

At recruitment, participants were asked how often they drank alcohol with the possible responses being: "daily or almost daily", "three or four times a week", "once or twice a week", "one to three times a month", "special occasions only", "never", or "prefer not to answer". Participants were also asked about their weekly and monthly consumption of alcoholic beverages, specifically: pints of beer, glasses of red wine, glasses of white wine/champagne, glasses of fortified wine, measures of spirits/liqueurs and glasses of other alcohol. A pint of beer was assumed to contain 20 grams of alcohol, and all other drinks were assigned 10 grams of alcohol. These were then summed to obtain the total weekly and monthly consumption of alcohol accordingly. We used participants reported weekly consumption of alcohol, if this was unknown (due to the participant reporting 'do not know' or 'prefer not to answer' for one or more of the relevant questions, except for 'other alcohol') we used monthly consumption, if available. Estimated daily totals were estimated by dividing weekly consumption by 7 (or monthly consumption by 30.4375). Alcohol consumption was categorised as <1 g/day, 1-9.99 g/day, 10-19.99 g/day, and ≥20 g/day, non-drinkers, or unknown. For participants who had unknown grams/day of alcohol but reported to consume alcohol on 'special occasions' were assigned to the category of '<1 g/day'. If the participant reported 'do not know' or 'prefer not to answer' to the questions on weekly or monthly consumption, they were coded as unknown.

Physical activity

Physical activity was determined from questions on the touchscreen questionnaire which asked about walking, moderate physical activity, and vigorous physical activity. These were used to estimate excess metabolic equivalent (MET)-hours/week of physical activity during work and leisure time. For each of the three activity categories (walking, moderate physical activity, and vigorous physical activity), participants were asked how many days in a typical week they did each of the activities for 10 minutes or more. For each category, participants who entered one or more days were then asked how many minutes they spent doing those activities on a typical day. For each activity category, the number of reported days was multiplied by the number of reported minutes on a typical day to generate duration of activity in minutes per week. Activity on a typical day of 1260 min per week (equivalent to an average of 3 hours per day) were truncated at 1260. Total MET values for each category from the International Physical Activity Questionnaire short form were: 3.3 for walking, 4.0 for moderate physical activity and 8.0 for vigorous physical activity. Excess MET values were therefore 2.3 for walking, 3.0 for moderate physical activity and 7.0 for vigorous physical activity. Excess MET-hours per week were calculated by multiplying the excess MET value for each activity by the duration of activity in hours per week.

Townsend deprivation index

Townsend deprivation index was based on the preceding national census output areas. Each participant was assigned a score in correspondence to the output area in which their postcode was located. From this, participants were spilt into quintiles from most deprived to least deprived and a missing category where postcode information was not provided.

Smoking status

Smoking was determined from questions from the touchscreen questionnaire at recruitment. Participants were asked “Do you smoke tobacco now?” and “in the past, how often have you smoked tobacco?” to determine their smoking status. Smokers were further divided based on how many cigarettes they said they smoked on average per day from the question ‘About how many cigarettes do you smoke on average each day?’.

Ethnicity

Ethnicity of participants was determined from questions in the touchscreen questionnaire ‘What is your ethnic group?’. Options included: white, mixed, Asian or Asian British, Black or Black British, Chinese, and other ethnic group. Participants that answered that they were Asian or Asian British were asked if they were “Indian, Pakistani, Bangladeshi or any other Asian background”. Participants who responded they were Indian, Pakistani, or Bangladeshi, were categorised into one group where those who responded as other Asian background were categorised into another group.

Education

For education, participants were asked ‘Which of the following qualification do you have?’ being able to select more than one. Possible answers were: College or University degree; A levels/AS levels or equivalent; O levels/ General Certificate of Secondary Education (GCSEs) or equivalent; Certificate of Secondary Education (CSEs) or equivalent; National vocational qualification (NVQ) or Higher National Diploma (HND) or Higher National Certificate (HNC) or equivalent; Other professional qualifications example: nursing, teaching; None of the above; Prefer not to answer. We grouped participants into the following categories, based on their highest reported level of education: (College or

University degree, vocational qualifications (other professional qualifications/NVQ or HND or HNC), optional national exams at ages 17 to 18 years (A levels/AS levels), national exams at age 16 years (O levels/GCSEs/CSEs), none of the above, unknown (prefer not to answer)).

Diabetes

Participants' diabetes status was determined using multiple variables from recruitment. First, from the question asked to participants 'Has a doctor ever told you that you have diabetes?' participants were classified as 'diabetic, 'not diabetic' or 'unknown' based on their response. As well, participants who reported to using metformin or insulin at recruitment were considered diabetic and included in the 'diabetic' category. Finally, if a participant had a measured glycated hemoglobin (HbA1c) of ≥ 48 mmol/mol at recruitment, they were defined as being diabetic and included in the 'diabetic' category.

Hormone replacement therapy and oral contraceptives

For women, use of hormone replace therapy (HRT) and oral contraceptives (OC) were categorised as 'current user', 'former user' and 'never user' or 'unknown/missing' based on the questions asked about HRT and OC use in the touchscreen recruitment questionnaire. All men were put into a separate category for all women-specific covariates. Women were asked 'Have you ever used hormone replacement therapy?' and if they answered yes: 'How old were you when you last used HRT?'. Women were asked to enter their age when they last used HRT or could choose 'Still taking HRT', or they could select 'prefer not to answer' or 'do not know'. Similarly, for OC, women were asked: 'Have you ever taken the contraceptive pill?' and if they answered yes were then asked: 'How old were you when you last used the contraceptive pill?'. Women were asked to enter their age when they last used OC or could

choose ‘Still taking OC’, or they could select ‘prefer not to answer’ or ‘do not know’. From this, women were categorised accordingly.

Menopausal status

Menopausal status was determined by multiple questions asked in the touchscreen questionnaire at recruitment. Women were defined as pre-menopausal if they:

- Answered ‘no’ to having gone through menopause, or
- Said they were ‘not sure’ or did not answer if they had gone through menopause and:
    - Were <50 years of age, did not have a bilateral oophorectomy/hysterectomy, and said they were not using HRT.
    - Were <50 years of age, said they were menstruating today, and did not have a bilateral oophorectomy/hysterectomy.

Women were defined as post-menopausal if they:

- Answered ‘yes’ to having gone through menopause
- Answered ‘not sure’ or did not answer if they had gone through menopause and:
    - were ≥55 years of age, or
    - had a bilateral oophorectomy

Women were defined as their menopausal status being unknown if:

- Answered ‘no’ to having gone through menopause and:
    - Did not answer no to using HRT, or
    - Did not answer no to having a bilateral oophorectomy, or
    - Did not answer no to having a hysterectomy, or
    - Were 50-54.9 years of age.

References

1. Swan G, Dodhia S, Farron-Wilson M, Powell N, Bush M. Food composition data and public health. Nutrition Bulletin. 2015;40(3):223-6.

2. Perez-Cornago A, Pollard Z, Young H, van Uden M, Andrews C, Piernas C, et al. Description of the updated nutrition calculation of the Oxford WebQ questionnaire and comparison with the previous version among 207,144 participants in UK Biobank. 2021. Eur J Nutr. In press.

3. Elliott P, Peakman TC, on behalf of UK Biobank. The UK Biobank sample handling and storage protocol for the collection, processing and archiving of human blood and urine. International Journal of Epidemiology. 2008;37(2):234-44.

Supplementary Tables

Supplementary Table S1. List of medications and UK Biobank codes from baseline used to exclude participants taking medication which may modify insulin-like growth factor-I concentrations

Medication code	Medication
1140868644	somatropin
1140857838	somatrem
1140857840	somatonorm 4iu injection
1141167490	somatuline la 30mg injection (pdr)+diluent+syringe
1140857748	genotropin 12iu multidose injection
1140857750	somatropin 12iu injection
1140868646	humatrope(rbe) 4iu(1.3mg) injection (pdr for recon)+diluent
1140868648	saizen(rmc) 4iu(1.33mg) injection (pdr for recon)+diluent
1140868650	norditropin(epr) 12iu(4mg) injection (pdr for recon)+diluent
1140884544	leuprorelin
1141157394	goserelin product
1140870194	goserelin
1140870196	zoladex 3.6 mg implant
1140921100	triptorelin
1141189852	decapeptyl sr 3mg injection (pdr for recon)+diluent
1141189772	gonapeptyl depot 3.75mg inj (pdr for recon)+solv p/f syringe
1140870248	buserelin
1141157392	buserelin product
1140870252	suprefact 100micrograms nasal spray
1140868490	gestanin 5mg tablet
1140870084	depostat 200mg/2ml oily injection
1140876638	cyproterone acetate+ethinyloestradiol
1141192344	cyproterone acetate+ethinylestradiol
1140884634	cyproterone
1140868524	androcur 50mg tablet
1140869270	medroxyprogesterone
1141190580	conjugated oestrogens 0.3mg / medroxyprogesterone 1.5mg tab
1140864232	provera 2.5mg tablet
1140857620	depo-provera 50mg/1ml injection
1140870274	flutamide
1140917306	bicalutamide
1140917310	casodex 50mg tablet
1141179886	propecia 1mg tablet
1140928222	andropatch 2.5mg/24hours transdermal patch
1140910802	androstanazol
1141193272	testogel 50mg gel 5g sachet
1141166354	testoderm 6mg/24hours transdermal patch
1140868534	primoteston depot 250mg/1ml oily injection
1140868536	restandol 40mg capsule
1140868538	sustanon 100 oily injection

1140864502	testotop tts 15mg transdermal patch
1141167430	lanreotide
1140870200	octreotide
1140870208	sandostatin 50micrograms/1ml injection
1141195128	pegvisomant
1141195032	somavert 10mg injection (pdr for recon) +solvent
1140857656	methyltestosterone product
1140857668	virormone-oral 5mg tablet
1140857736	virormone 10mg/1ml injection
1140865136	yohimbine/pemoline/methyltestosterone
1140868532	testosterone product
1140868528	pro-viron 25mg tablet
1140868526	mesterolone
1140868524	androcur 50mg tablet
1140868534	primoteston depot 250mg/1ml oily injection
1140868536	restandol 40mg capsule
1140868538	sustanon 100 oily injection
1140868550	finasteride
1140868608	proscar 5mg tablet
1140868614	deca-durabolin 25mg/1ml oily injection
1140868618	stanozolol
1140868620	stromba 5mg tablet
1141179886	propecia 1mg tablet
1141192000	dutasteride
1141192004	avodart 500micrograms capsule
1140868968	danazol
1140870284	prostap sr 3.75mg injection (pdr for recon)+diluent+kit
1141201718	nebido 1000mg/4ml solution for injection
1140923018	anastrozole
1141171100	exemestane
1141145896	letrozole
1140870164	tamoxifen
1140888684	diazoxide
1140928276	humatrope(rbe) 18iu(6mg) inj cartridge(pdr for recon)+dil
1141189090	rosiglitazone 1mg / metformin 500mg tablet
1140874686	glucophage 500mg tablet
1141190802	nutropinaq 10mg(30iu)/2ml injection cartridge
1140923890	zomacton(rbe) 12iu(4mg) injection (pdr for recon)+diluent
1140868810	geref 50 injection (pdr for recon)+solvent
1140909918	biosynthetic human growth hormone
1140882976	growth hormone product

Supplementary Table S2. Geometric mean difference and percentage change of IGF-I **comparing the highest quintile to the lowest quintile** of nutrients using the baseline IGF-I measurement and in secondary analyses using the follow-up IGF-I measurement ~4 years after recruitment

	WebQ subsample (N=11,815)		Secondary analysis: WebQ repeat IGF-I sample (N=2,724)	
	**Absolute change of IGF-I (nmol/L; 95% CI)**	**Percentage change of IGF-I (95% CI)**	**Absolute change of IGF-I (nmol/L; 95% CI)**	**Percentage change of IGF-I (95% CI)**
Total protein	**1.72 (1.40 to 2.03)**	**8.45% (6.92% to 9.98%)**	**1.08 (0.44 to 1.73)**	**5.46% (2.19% to 8.73%)**
Protein from plant sources	0.89 (0.58 to 1.20)	4.32% (2.84% to 5.80%)	0.56 (-0.07 to 1.20)	2.80% (-0.37% to 5.98%)
Protein from animal sources	**1.19 (0.88 to 1.50)**	**5.77% (4.26% to 7.27%)**	0.63 (-0.01 to 1.28)	3.15% (-0.03% to 6.34%)
Protein from dairy sources	0.95 (0.64 to 1.25)	4.59% (3.12% to 6.06%)	**1.39 (0.75 to 2.04)**	**6.98% (3.75% to 10.20%)**
Protein from milk	**1.18 (0.87 to 1.49)**	**5.72% (4.23% to 7.21%)**	**1.38 (0.73 to 2.02)**	**6.90% (3.67% to 10.14%)**
Protein from yogurt	0.69 (0.41 to 0.96)	3.28% (1.98% to 4.58%)	0.95 (0.38 to 1.52)	4.73% (1.90% to 7.55%)
Protein from cheese	0.00 (-0.31 to 0.30)	-0.02% (-1.45% to 1.42%)	0.00 (-0.64 to 0.64)	0.00% (-3.12% to 3.11%)
Protein from animal products (not dairy)	0.65 (0.34 to 0.95)	3.10% (1.62% to 4.58%)	0.05 (-0.59 to 0.69)	0.25% (-2.90% to 3.40%)
Total carbohydrate	0.48 (0.17 to 0.80)	2.33% (0.80% to 3.85%)	0.57 (-0.10 to 1.24)	2.79% (-0.50% to 6.09%)
Total sugars	0.09 (-0.22 to 0.40)	0.42% (-1.05% to 1.88%)	0.07 (-0.58 to 0.72)	0.33% (-2.82% to 3.48%)
Free sugars	-0.96 (-1.27 to -0.66)	-4.46% (-5.87% to -3.05%)	-0.04 (-0.69 to 0.60)	-0.21% (-3.34% to 2.92%)
Non-free sugars	1.02 (0.71 to 1.33)	4.96% (3.45% to 6.48%)	0.84 (0.18 to 1.49)	4.18% (0.92% to 7.44%)
Total starch	0.41 (0.10 to 0.72)	1.95% (0.47% to 3.42%)	0.64 (0.00 to 1.29)	3.18% (-0.02% to 6.37%)
Starch from wholegrains	**1.08 (0.77 to 1.39)**	**5.23% (3.74% to 6.71%)**	**1.26 (0.63 to 1.90)**	**6.41% (3.19% to 9.63%)**
Starch from refined grains	0.29 (-0.01 to 0.60)	1.39% (-0.06% to 2.84%)	0.16 (-0.49 to 0.80)	0.77% (-2.35% to 3.89%)
Fibre	**1.32 (1.00 to 1.65)**	**6.21% (4.87% to 7.83%)**	**1.11 (0.39 to 1.82)**	**5.26% (1.98% to 8.76%)**
Total fat	-0.54 (-0.85 to -0.23)	-2.51% (-3.95% to -1.07%)	-0.61 (-1.26 to 0.05)	-2.89% (-6.00% to 0.22%)
Fat from plant sources	-0.07 (-0.37 to 0.24)	-0.32% (-1.76% to 1.13%)	-0.11 (-0.76 to 0.53)	-0.55% (-3.68% to 2.58%)
Fat from animal sources	-0.37 (-0.67 to -0.07)	-1.73% (-3.16% to -0.30%)	-0.17 (-0.81 to 0.47)	-0.82% (-3.92% to 2.28%)
Fat from dairy sources	-0.17 (-0.47 to 0.13)	-0.81% (-2.24% to 0.62%)	0.10 (-0.54 to 0.75)	0.50% (-2.61% to 3.61%)
Fat from milk	0.48 (0.18 to 0.79)	2.28% (0.83% to 3.73%)	0.66 (0.02 to 1.30)	3.22% (0.09% to 6.35%)
Fat from yogurt	0.50 (0.23 to 0.77)	2.39% (1.10% to 3.67%)	1.00 (0.43 to 1.57)	4.97% (2.15% to 7.80%)
Fat from cheese	-0.15 (-0.46 to 0.15)	-0.72% (-2.15% to 0.71%)	0.13 (-0.51 to 0.78)	0.65% (-2.47% to 3.77%)
Fat from animal products (not dairy)	-0.25 (-0.56 to 0.05)	-1.19% (-2.62% to 0.24%)	-0.15 (-0.79 to 0.49)	-0.74% (-3.84% to 2.36%)

Alcohol (*comparing >40 g/day to <1 g/day)*	**-1.36 (-1.00 to -1.71)**	**-6.32% (-4.68% to -7.97%)**	**-0.50 (-1.24 to 0.24)**	**-2.43% (-6.05% to 1.18%)**
Total energy	0.25 (-0.05 to 0.55)	1.19% (-0.26% to 2.64%)	0.52 (-0.62 to 1.65)	2.55% (-3.06% to 8.17%)
Total energy (not from alcohol)	0.55 (0.24 to 0.85)	2.63% (1.18% to 4.09%)	0.71 (-0.43 to 1.84)	3.53% (-2.14% to 9.21%)

All models are adjusted for sex, age at recruitment, region of recruitment, body mass index, height, physical activity, Townsend deprivation index, education, smoking, alcohol consumption, ethnicity, diabetes, energy intake (except when energy intake was the exposure of interest), and women specific covariates: hormone replacement therapy use, oral contraceptive use, and menopausal status.

For baseline IGF-I measurements, quintiles of percentage of energy from carbohydrate sources, percentage of energy from fat sources, and percentage of protein sources, fibre, alcohol categories, and energy intake quintiles calculated from a minimum of four (maximum of five) averaged 24-hour web-based dietary assessments with one assessment completed at recruitment for baseline IGF-I measurement when blood sample was provided. For follow-up IGF-I measurements, quintiles of percentage of energy from carbohydrates, fat sources, protein sources, and intake of fibre, alcohol categories, and energy quintiles calculated from a minimum of four (maximum of five) averaged 24-hour web-based dietary assessments completed at any time.

Percentages and geometric mean difference from highest category or quintile compared to lowest category or quintile of intake are from Figures 1, and 2 in main text for baseline IGF-I measurement. Quintiles for follow-up IGF-I measurement from highest category or quintile to lowest category. All quintiles of nutrients are not presented for follow-up measurement of IGF-I.

Bolded nutrients represent a ≥5% difference in IGF-I concentrations comparing the highest category or quintile to the lowest category or quintile.

Abbreviations: CI, confidence interval; IGF-I, insulin-like growth factor-I

Supplementary Table S3. Macronutrients and fibre from the 24-hour dietary assessment subsample ranked by largest baseline IGF-I concentration **percentage difference between highest and lowest category and by 2.5% energy increase for macronutrients and 5 g/day increase for fibre**

Food or nutrient	Geometric mean difference between highest versus lowest category/quintile (% change)	Geometric mean difference per 2.5% energy for macronutrients or 5 g/day for fibre (% change)
**Total protein (percentage of energy)**	**8.45% (6.92% to 9.98%)**	**2.69% (2.24% to 3.14%)**
**Protein from yogurt (percentage of energy)**	**3.28% (1.98% to 4.58%)**	**6.35% (3.90% to 8.80%)**
**Alcohol (categories)**	**-6.32% (-4.68% to -7.97%)**	**-**
**Fibre (grams)**	**6.21% (4.87% to 7.83%)**	**2.12% (1.60% to 2.64%)**
**Protein from animal sources (percentage of energy)**	**5.77% (4.26% to 7.27%)**	**1.62% (1.22% to 2.02%)**
**Protein from milk (percentage of energy)**	**5.72% (4.23% to 7.21%)**	**5.68% (4.27% to 7.08%)**
**Starch from wholegrains (percentage of energy)**	**5.23% (3.74% to 6.71%)**	**1.15% (0.84% to 1.45%)**
Non-free sugars (percentage of energy)	4.96% (3.45% to 6.48%)	0.85% (0.61% to 1.09%)
Protein from dairy sources (percentage of energy)	4.59% (3.12% to 6.06%)	3.47% (2.51% to 4.43%)
Free sugars (percentage of energy)	-4.46% (-5.87% to -3.05%)	-0.94% (-1.20% to -0.68%)
Protein from plant sources (percentage of energy)	4.32% (2.84% to 5.80%)	2.60% (1.68% to 3.51%)
Protein from animal products (not dairy; percentage of energy)	3.10% (1.62% to 4.58%)	1.04% (0.63% to 1.44%)
Fat from yogurt (percentage of energy)	2.39% (1.10% to 3.67%)	2.65% (0.47% to 4.83%)
Total energy (not from alcohol; quintiles)	2.63% (1.18% to 4.09%)	-

All models are adjusted for sex, age at recruitment, region of recruitment, body mass index, height, physical activity, Townsend deprivation index, education, smoking, alcohol consumption, ethnicity, diabetes, energy intake (except when energy intake was the exposure of interest), and women specific covariates: hormone replacement therapy use, oral contraceptive use, and menopausal status.

Quintiles of percentage of energy from carbohydrate sources, percentage of energy from fat sources and percentage of protein sources, fibre, alcohol categories, and energy intake quintiles calculated from a minimum of four (maximum of five) averaged 24-hour web-based dietary assessments with one assessment completed at recruitment.

Percentage difference from highest category or quintile compared to lowest category or quintile of intake are from Figures 1, 2, and 3 in main text for baseline IGF-I measurement.

Bolded nutrients represent the top 10 associations with IGF-I concentrations from the baseline measurement.

Abbreviations: CI, confidence interval; g/day, grams per day; IGF-I, insulin-like growth factor-I.

Supplementary Table S4. Macronutrient **per 2.5% of energy intake** in association with absolute difference and percentage change of IGF-I for the WebQ 24-hour dietary assessment subsample (N=11,815) and in secondary analysis for participants who completed four or more WebQs and had IGF-I measured ~4 years after recruitment (N=2,724)

	**WebQ subsample[1] (N=11,815)**		**Secondary analysis: Four or more completed WebQs and follow-up IGF-I measurement (N=2,724)[2]**	
**Per 2.5% energy increase**	**Absolute change of IGF-I (nmol/L; 95% CI)**	**Percentage change of IGF-I (95% CI)**	**Absolute change of IGF-I (nmol/L; 95% CI)**	**Percentage change of IGF-I (95% CI)**
Total protein	0.56 (0.47 to 0.66)	2.69% (2.24% to 3.14%)	0.31 (0.11 to 0.51)	1.71% (0.74% to 2.67%)
Protein from plant sources	0.56 (0.36 to 0.76)	2.60% (1.68% to 3.51%)	0.28 (-0.11 to 0.67)	1.38% (-0.51% to 3.27%)
Protein from animal sources	0.34 (0.25 to 0.42)	1.62% (1.22% to 2.02%)	0.18 (0.00 to 0.35)	1.00% (0.17% to 1.83%)
Protein from dairy sources	0.73 (0.52 to 0.94)	3.47% (2.51% to 4.43%)	0.88 (0.46 to 1.30)	4.44% (2.42% to 6.46%)
Protein from milk	**1.20 (0.90 to 1.51)**	**5.68% (4.27% to 7.08%)**	**1.42 (0.81 to 2.03)**	**7.49% (4.54% to 10.44%)**
Protein from yogurt	**1.33 (0.79 to 1.86)**	**6.35% (3.90% to 8.80%)**	**0.99 (0.06 to 2.05)**	**5.59% (0.53% to 10.65%)**
Protein from cheese	-0.07 (-0.40 to 0.25)	-0.34% (-1.84% to 1.16%)	0.16 (-0.49 to 0.82)	0.21% (-2.94% to 3.37%)
Protein from animal products (not dairy)	0.21 (0.13 to 0.30)	1.04% (0.63% to 1.44%)	0.03 (-0.15 to 0.20)	0.26% (-0.59% to 1.10%)
Total carbohydrate	0.07 (0.03 to 0.11)	0.33% (0.14% to 0.52%)	0.08 (0.00 to 0.16)	0.41% (0.00% to 0.81%)
Total sugars	0.02 (-0.03 to 0.06)	0.05% (-0.15% to 0.26%)	0.03 (-0.06 to 0.12)	0.14% (-0.28% to 0.55%)
Free sugars	-0.19 (-0.25 to -0.13)	-0.94% (-1.20% to -0.68%)	-0.02 (-0.14 to 0.09)	-0.19% (-0.74% to 0.36%)
Non-free sugars	0.18 (0.13 to 0.23)	0.85% (0.61% to 1.09%)	0.06 (-0.04 to 0.16)	0.34% (-0.15% to 0.83%)
Total starch	0.09 (0.04 to 0.14)	0.44% (0.20% to 0.67%)	0.08 (-0.03 to 0.18)	0.42% (-0.07% to 0.92%)
Starch from wholegrains	0.24 (0.17 to 0.30)	1.15% (0.84% to 1.45%)	0.25 (0.12 to 0.38)	1.26% (0.64% to 1.87%)
Starch from refined grains	0.05 (0.00 to 0.11)	0.25% (0.00% to 0.50%)	0.00 (-0.10 to 0.11)	-0.04% (-0.56% to 0.48%)
Fibre (per 5 grams/day)	0.46 (0.35 to 0.57)	2.12% (1.60% to 2.64%)	0.23 (0.01 to 0.45)	1.19% (0.12% to 2.27%)
Total fat	-0.10 (-0.14 to -0.05)	-0.44% (-0.67% to -0.21%)	-0.07 (-0.18 to 0.03)	-0.42% (-0.91% to 0.08%)
Fat from plant sources	-0.04 (-0.10 to 0.03)	-0.19% (-0.47% to 0.10%)	-0.04 (-0.17 to 0.08)	-0.26% (-0.86% to 0.34%)
Fat from animal sources	-0.08 (-0.13 to -0.03)	-0.34% (-0.57% to -0.11%)	-0.04 (-0.15 to 0.06)	-0.23% (-0.72% to 0.25%)
Fat from dairy sources	-0.07 (-0.14 to 0.01)	-0.33% (-0.67% to 0.01%)	0.00 (-0.15 to 0.15)	-0.13% (-0.85% to 0.58%)
Fat from milk	0.29 (0.06 to 0.52)	1.28% (0.22% to 2.34%)	0.47 (0.01 to 0.93)	2.60% (0.39% to 4.81%)
Fat from yogurt	0.53 (0.05 to 1.00)	2.65% (0.47% to 4.83%)	**0.90 (0.01 to 1.80)**	**5.02% (0.63% to 9.40%)**

Fat from cheese	-0.10 (-0.22 to 0.03)	-0.46% (-1.05% to 0.13%)	0.11 (-0.15 to 0.37)	0.33% (-0.92% to 1.57%)
Fat from animal products (not dairy)	-0.07 (-0.13 to -0.01)	-0.28% (-0.57% to 0.01%)	-0.06 (-0.19 to 0.06)	-0.26% (-0.86% to 0.34%)

All models are adjusted for sex, age at recruitment, region of recruitment, body mass index, height, physical activity, Townsend deprivation index, education, smoking, alcohol consumption, ethnicity, diabetes, energy intake, and women specific covariates: hormone replacement therapy use, oral contraceptive use, and menopausal status.

[1] WebQ 24-hour dietary assessment subsample represents a 2.5% energy change averaged across a minimum of four (maximum of five) 24-hour web-based diet assessments with one assessment completed at recruitment with baseline IGF-I concentrations. Fibre intake represents a 5 grams per day increase.

[2] Analysis assessing macronutrients and fibre intake calculated for participants with a follow-up IGF-I measurement. Macronutrients and fibre intake were calculated by using a minimum of four (maximum of five) averaged 24-hour web-based dietary assessments completed at any time. IGF-I was measured after all completed WebQs (mean follow-up time from last WebQ to follow-up blood measurement: 0.64 years, mean follow-up from recruitment to follow-up blood measurement: 4.3 years)

Bolded macronutrients represent ≥5% difference of IGF-I concentrations from the follow-up measurement

Abbreviations: CI, confidence intervals; g/day, grams per day; IGF-I, insulin-like growth factor-I.

Supplementary Figures

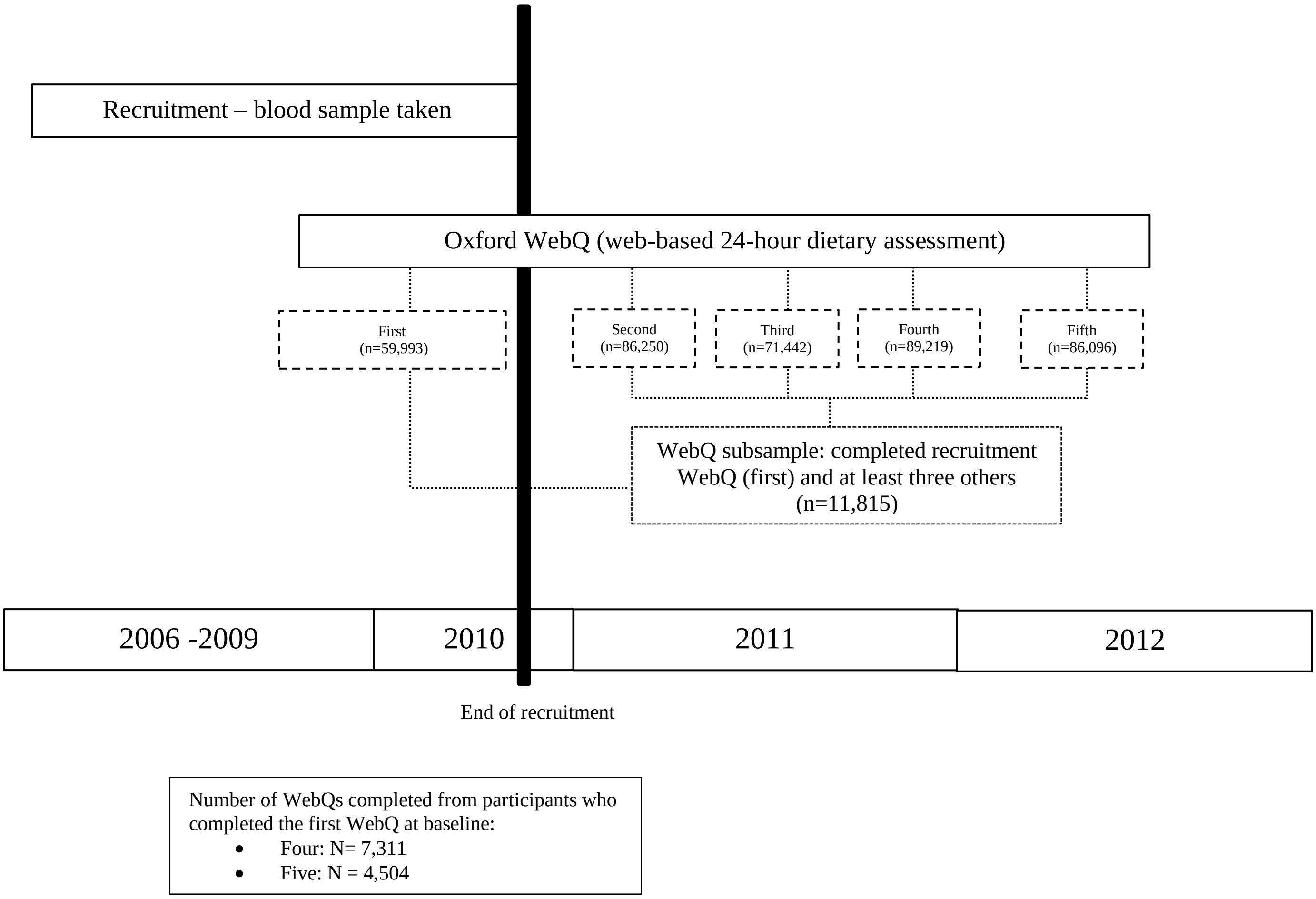

Supplementary Figure S1. Dietary assessment for participants in the UK Biobank over time

Numbers for 24-hour dietary assessments (WebQs) exclude participants who withdrew consent, were diagnosed with a prevalent cancer at recruitment, were taking potentially altering IGF-I medications, did not have a measured value for IGF-I concentration at recruitment or the 24-hour dietary assessment was deemed invalid due to reporting extreme energy intake or participant said they were ill or fasting on the respective day.

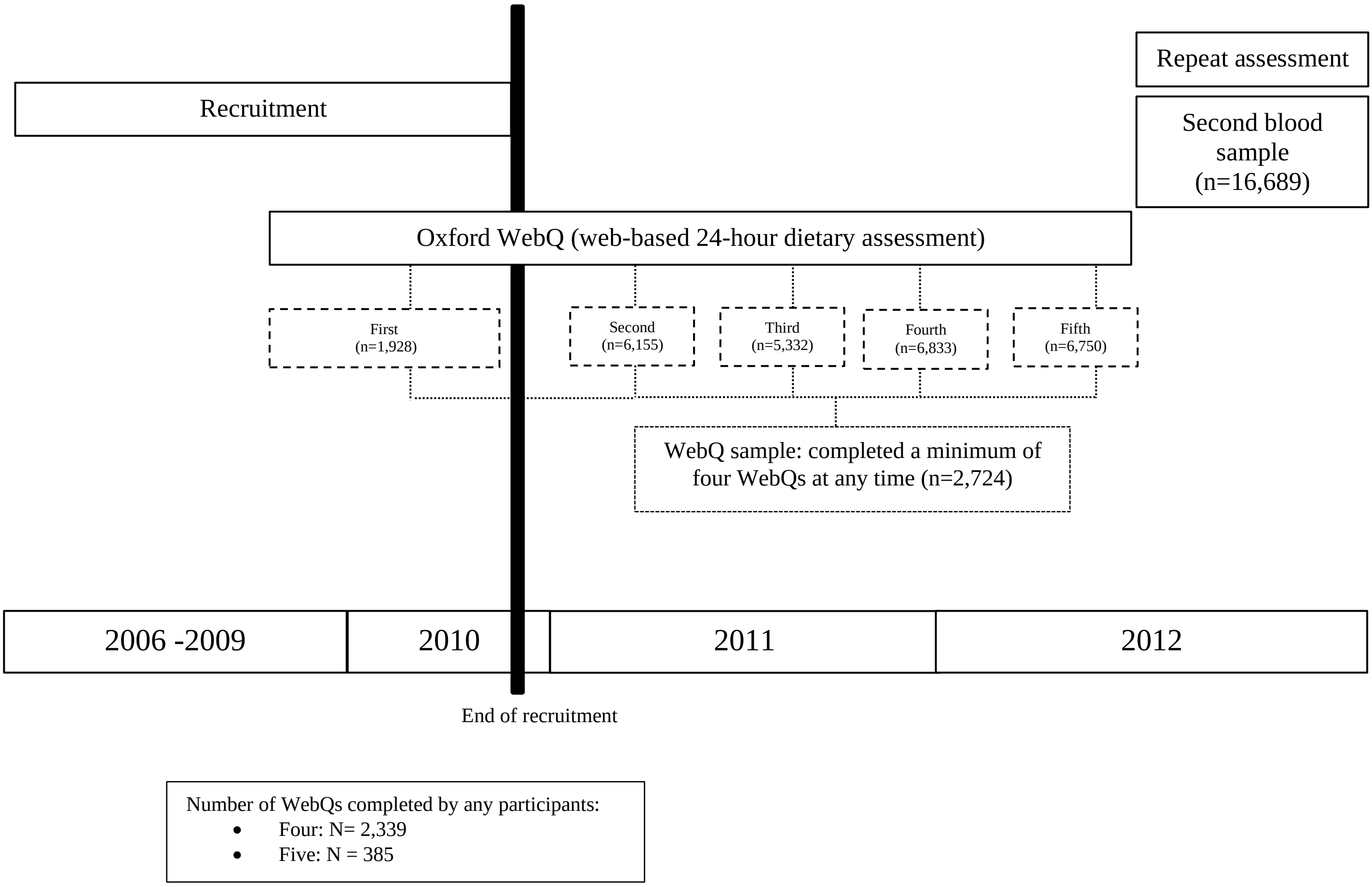

Supplementary Figure S2. Dietary assessment for participants **with follow-up blood sample** in the UK Biobank over time

Numbers for 24-hour dietary assessments (WebQs) exclude participants who withdrew their consent, were diagnosed with a prevalent cancer at recruitment or were taking potentially altering IGF-I medications, did not have a measured value for the follow-up IGF-I measurement or the 24-hour dietary assessment was deemed invalid due to reporting extreme energy intake or participant said they were ill or fasting on the respective day.

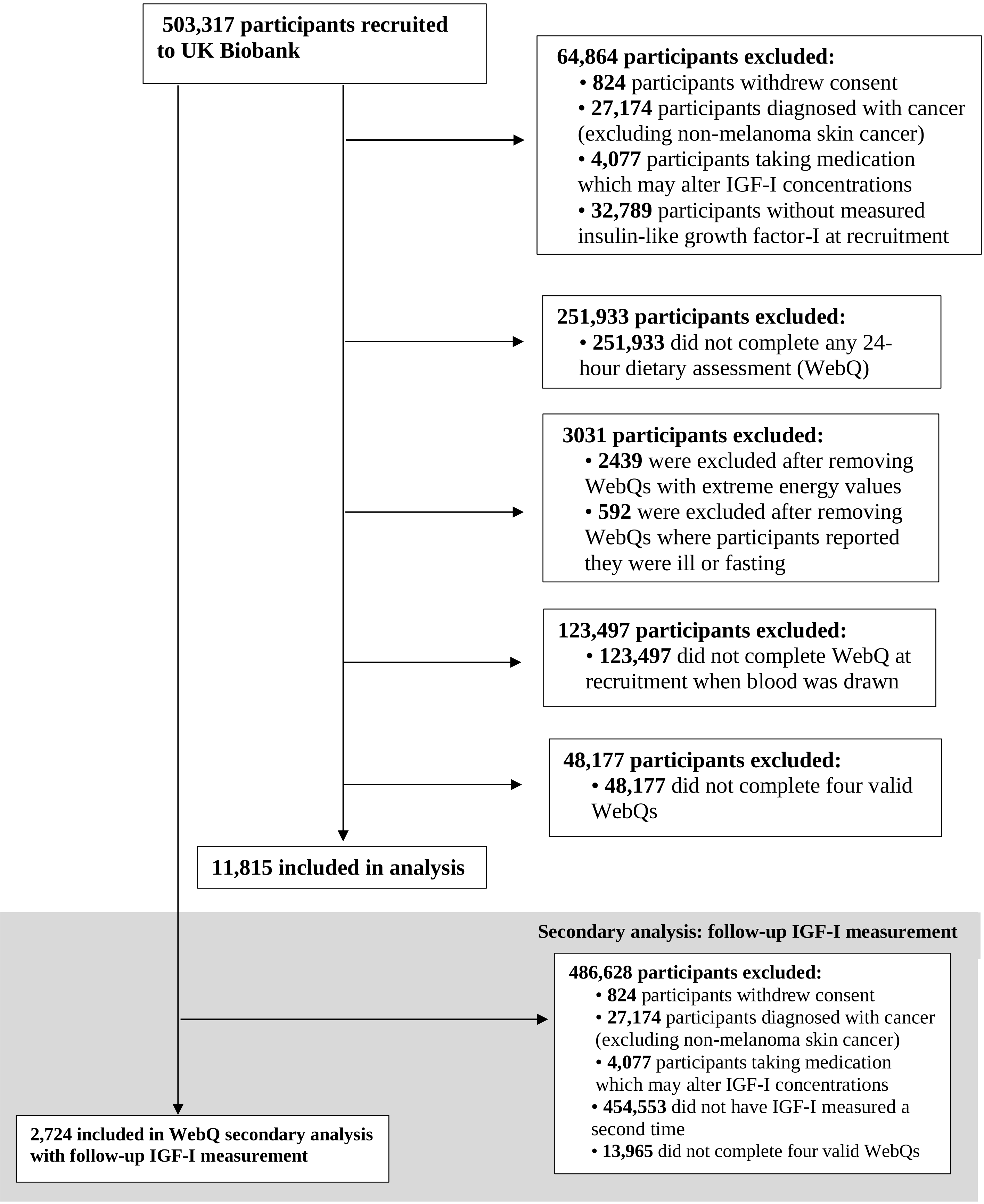

Supplementary Figure S3. Flow chart of exclusion criteria for WebQ 24-hour dietary assessment subsample, and secondary analysis restricting to participants who had a follow-up IGF-I measurements

WebQ subsample	N	Mean g/day	Percentage of energy	Geometric mean IGF-I concentration (nmol/L)
**Total protein**				
Q1 (Lowest)	2,364	68.1	12.51%	20.42 (20.22 - 20.64)
Q2	2,363	76.9	14.46%	21.14 (20.92 - 21.36)
Q3	2,363	81.3	15.77%	21.50 (21.28 - 21.72)
Q4	2,363	84.5	17.17%	21.37 (21.15 - 21.59)
Q5 (Highest)	2,362	89.8	19.89%	21.74 (21.51 - 21.96)
**Protein from plant sources**				
Q1 (Lowest)	2,364	21.5	4.08%	20.44 (20.23 - 20.65)
Q2	2,363	25.4	4.89%	21.07 (20.85 - 21.29)
Q3	2,363	27.7	5.44%	21.58 (21.35 - 21.80)
Q4	2,363	30.4	6.07%	21.54 (21.32 - 21.76)
Q5 (Highest)	2,362	36.5	7.53%	21.54 (21.32 - 21.77)
**Protein from animal sources**				
Q1 (Lowest)	2,364	33.8	6.35%	20.78 (20.57 - 21.00)
Q2	2,363	47.6	8.92%	21.10 (20.88 - 21.31)
Q3	2,363	53.3	10.29%	21.54 (21.32 - 21.76)
Q4	2,363	58.2	11.73%	21.21 (20.99 - 21.43)
Q5 (Highest)	2,362	66.2	14.51%	21.52 (21.30 - 21.75)
**Protein from dairy sources**				
Q1 (Lowest)	2,364	5.8	1.12%	20.54 (20.33 - 20.75)
Q2	2,363	10.5	2.04%	21.02 (20.81 - 21.24)
Q3	2,363	13.3	2.62%	21.42 (21.20 - 21.64)
Q4	2,363	15.9	3.23%	21.54 (21.32 - 21.76)
Q5 (Highest)	2,362	20.9	4.44%	21.64 (21.42 - 21.86)
**Protein from milk**				
Q1 (Lowest)	2,364	1.4	0.28%	20.54 (20.33 - 20.75)
Q2	2,363	5	0.96%	20.81 (20.59 - 21.02)
Q3	2,363	7.1	1.38%	21.48 (21.26 - 21.70)
Q4	2,363	8.8	1.79%	21.54 (21.32 - 21.76)
Q5 (Highest)	2,362	11.6	2.57%	21.81 (21.58 - 22.03)
**Protein from yogurt**				
Q1 (Lowest)	4,247	0	0.00%	20.83 (20.67 - 20.99)
Q2	715	1	0.18%	21.07 (20.67 - 21.48)
Q3	2,128	1.5	0.30%	21.31 (21.08 - 21.55)
Q4	2,363	2.8	0.57%	21.53 (21.31 - 21.75)
Q5 (Highest)	2,362	5.2	1.16%	21.63 (21.41 - 21.85)
**Protein from cheese**				
Q1 (Lowest)	2,364	0.2	0.04%	21.12 (20.90 - 21.34)
Q2	2,363	2.1	0.40%	21.23 (21.01 - 21.45)
Q3	2,363	3.8	0.74%	21.29 (21.07 - 21.51)
Q4	2,363	6	1.16%	21.26 (21.04 - 21.48)
Q5 (Highest)	2,362	10.3	2.06%	21.23 (21.02 - 21.46)
**Protein from animal products (not dairy)**				
Q1 (Lowest)	2,364	19.6	3.65%	20.96 (20.74 - 21.18)
Q2	2,363	33.5	6.28%	21.35 (21.13 - 21.57)
Q3	2,363	39.8	7.65%	21.38 (21.16 - 21.60)
Q4	2,363	45.3	9.04%	21.28 (21.07 - 21.51)
Q5 (Highest)	2,362	54.5	11.73%	21.17 (20.95 - 21.39)

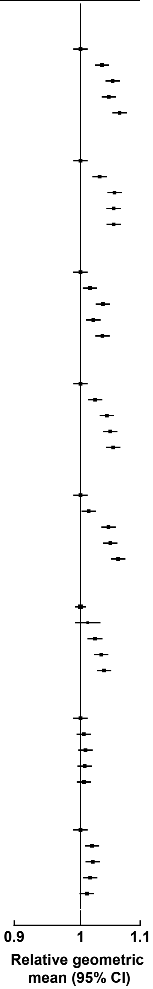

WebQ subsample	N	Mean g/day	Percentage of energy	Geometric mean IGF-I concentration (nmol/L)
**Total fat**				
Q1 (Lowest)	2,364	51.7	24.23%	21.35 (21.13 - 21.57)
Q2	2,363	64.5	28.81%	21.38 (21.16 - 21.61)
Q3	2,363	72.7	31.52%	21.30 (21.08 - 21.52)
Q4	2,363	80.8	34.27%	21.17 (20.95 - 21.39)
Q5 (Highest)	2,362	93.5	38.83%	20.94 (20.73 - 21.16)
**Fat from plant sources**				
Q1 (Lowest)	2,364	19.2	8.81%	20.90 (20.68 - 21.12)
Q2	2,363	26.2	11.62%	21.35 (21.13 - 21.57)
Q3	2,363	31.2	13.54%	21.51 (21.29 - 21.73)
Q4	2,363	36.3	15.64%	21.26 (21.04 - 21.48)
Q5 (Highest)	2,362	47.3	19.96%	21.13 (20.91 - 21.35)
**Fat from animal sources**				
Q1 (Lowest)	2,364	24.3	10.98%	21.40 (21.18 - 21.62)
Q2	2,363	34.1	14.95%	21.48 (21.26 - 21.70)
Q3	2,363	40.2	17.40%	21.21 (20.99 - 21.43)
Q4	2,363	46.7	20.02%	21.11 (20.90 - 21.33)
Q5 (Highest)	2,362	57.7	24.74%	20.94 (20.73 - 21.16)
**Fat from dairy sources**				
Q1 (Lowest)	2,364	3.6	1.61%	21.16 (20.95 - 21.38)
Q2	2,363	7.6	3.41%	21.27 (21.05 - 21.49)
Q3	2,363	11	4.88%	21.34 (21.12 - 21.57)
Q4	2,363	15.3	6.69%	21.30 (21.08 - 21.52)
Q5 (Highest)	2,362	24.7	10.69%	21.06 (20.85 - 21.28)
**Fat from milk**				
Q1 (Lowest)	2,364	0.3	0.13%	21.02 (20.80 - 21.24)
Q2	2,363	1.3	0.56%	21.04 (20.82 - 21.26)
Q3	2,363	2.7	1.17%	21.13 (20.91 - 21.35)
Q4	2,363	3.9	1.74%	21.33 (21.12 - 21.56)
Q5 (Highest)	2,362	6.1	2.90%	21.63 (21.40 - 21.85)
**Fat from yogurt**				
Q1 (Lowest)	4,247	0	0.00%	20.83 (20.67 - 20.99)
Q2	715	0.3	0.12%	20.90 (20.51 - 21.31)
Q3	2,128	0.5	0.23%	21.40 (21.16 - 21.63)
Q4	2,363	1.1	0.49%	21.63 (21.41 - 21.85)
Q5 (Highest)	2,362	2.6	1.21%	21.50 (21.28 - 21.73)
**Fat from cheese**				
Q1 (Lowest)	2,364	0.2	0.08%	21.15 (20.93 - 21.37)
Q2	2,363	2.3	0.98%	21.30 (21.08 - 21.52)
Q3	2,363	4.3	1.85%	21.24 (21.02 - 21.46)
Q4	2,363	6.8	2.95%	21.32 (21.10 - 21.54)
Q5 (Highest)	2,362	11.8	5.24%	21.13 (20.91 - 21.35)
**Fat from animal products (not dairy)**				
Q1 (Lowest)	2,364	15.4	6.77%	21.30 (21.08 - 21.52)
Q2	2,363	23	9.99%	21.35 (21.14 - 21.58)
Q3	2,363	28	11.99%	21.38 (21.16 - 21.60)
Q4	2,363	33	14.11%	21.20 (20.98 - 21.42)
Q5 (Highest)	2,362	41.5	17.97%	20.90 (20.69 - 21.12)

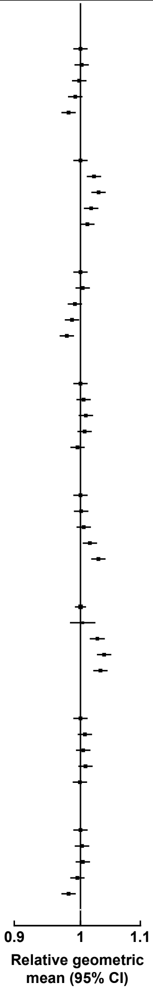

Supplementary Figure S4. Minimally adjusted models for percentage of energy intake from proteins and fats separated by quintiles in association with geometric mean concentrations of IGF-I in the WebQ 24-hour dietary assessment subsample (N=11,815)

All models are adjusted for sex and age at recruitment.

Supplementary Methods and Materials

Percentage of energy from protein and fat sources calculated from a minimum of four averaged 24-hour web-based diet assessments with one assessment completed at recruitment.

Grams calculated as mean per day within each quintile.

Percentage of energy calculated by mean percentage of energy per day in each quintile.

Abbreviations: CI, confidence intervals; g, grams; IGF-I, insulin-like growth factor-I; N, number of participants; Q, quintile.

WebQ subsample	N	Mean g/day	Percentage of energy	Geometric mean IGF-I concentration (nmol/L)
**Total carbohydrate**				
Q1 (Lowest)	2,364	205.4	39.68%	20.59 (20.37 - 20.80)
Q2	2,363	240.3	46.11%	21.34 (21.12 - 21.56)
Q3	2,363	253.4	49.59%	21.31 (21.09 - 21.53)
Q4	2,363	269.6	52.89%	21.51 (21.29 - 21.73)
Q5 (Highest)	2,362	284.1	58.45%	21.41 (21.19 - 21.63)
**Total sugar**				
Q1 (Lowest)	2,364	84.2	16.54%	20.81 (20.59 - 21.02)
Q2	2,363	108.7	21.14%	21.35 (21.13 - 21.57)
Q3	2,363	123.5	24.09%	21.38 (21.16 - 21.60)
Q4	2,363	137.2	27.19%	21.30 (21.08 - 21.52)
Q5 (Highest)	2,362	161.4	32.97%	21.31 (21.09 - 21.53)
**Total free sugars**				
Q1 (Lowest)	2,364	28.1	5.63%	21.40 (21.19 - 21.63)
Q2	2,363	44.6	8.67%	21.34 (21.12 - 21.56)
Q3	2,363	56.2	10.78%	21.43 (21.21 - 21.65)
Q4	2,363	68.9	13.14%	21.29 (21.08 - 21.52)
Q5 (Highest)	2,362	93.1	17.70%	20.68 (20.46 - 20.89)
**Total non-free sugar**				
Q1 (Lowest)	2,364	36.2	6.96%	20.31 (20.10 - 20.52)
Q2	2,363	52.3	10.19%	21.18 (20.96 - 21.40)
Q3	2,363	62.8	12.60%	21.47 (21.25 - 21.70)
Q4	2,363	75.2	15.29%	21.60 (21.38 - 21.83)
Q5 (Highest)	2,362	97.7	20.96%	21.61 (21.39 - 21.83)
**Total starch**				
Q1 (Lowest)	2,364	92.1	17.89%	20.83 (20.62 - 21.05)
Q2	2,363	117.5	22.46%	21.07 (20.86 - 21.29)
Q3	2,363	129.9	24.96%	21.22 (21.00 - 21.44)
Q4	2,363	140.9	27.44%	21.62 (21.40 - 21.84)
Q5 (Highest)	2,362	156.3	31.80%	21.40 (21.18 - 21.62)
**Starch from wholegrain**				
Q1 (Lowest)	2,364	3.9	0.75%	20.42 (20.21 - 20.64)
Q2	2,363	15.7	3.05%	21.11 (20.90 - 21.33)
Q3	2,363	25.2	4.93%	21.19 (20.97 - 21.41)
Q4	2,363	35.1	7.04%	21.54 (21.32 - 21.76)
Q5 (Highest)	2,362	53.7	11.27%	21.90 (21.68 - 22.13)
**Starch from refined grain**				
Q1 (Lowest)	2,364	5.4	1.02%	21.13 (20.91 - 21.35)
Q2	2,363	19.3	3.67%	21.18 (20.96 - 21.40)
Q3	2,363	31.2	5.99%	21.30 (21.08 - 21.52)
Q4	2,363	44.5	8.62%	21.31 (21.09 - 21.53)
Q5 (Highest)	2,362	67.6	13.78%	21.23 (21.01 - 21.45)
**Fibre**				
Q1 (Lowest)	2,364	11.5	-	20.34 (20.13 - 20.55)
Q2	2,363	15.1	-	21.07 (20.85 - 21.29)
Q3	2,363	17.6	-	21.34 (21.13 - 21.57)
Q4	2,363	20.3	-	21.58 (21.36 - 21.80)
Q5 (Highest)	2,362	25.8	-	21.83 (21.61 - 22.06)
**Alcohol**				
<1 grams/day	2,551	0.03	0.12%	21.24 (21.03 - 21.45)
1-9 grams/day	2,852	5.4	1.94%	21.49 (21.29 - 21.69)
10-19 grams/day	2,440	14.7	5.09%	21.56 (21.34 - 21.78)
20-39.9 grams/day	2,626	28.5	9.47%	21.27 (21.06 - 21.48)
40+ grams/day	1,346	56.6	17.36%	20.01 (19.73 - 20.29)
**Energy**				
Q1 (Lowest)	2,364	-	-	20.98 (20.77 - 21.20)
Q2	2,363	-	-	21.39 (21.17 - 21.61)
Q3	2,363	-	-	21.17 (20.95 - 21.39)
Q4	2,363	-	-	21.34 (21.12 - 21.56)
Q5 (Highest)	2,362	-	-	21.26 (21.04 - 21.48)
**Energy excluding alcohol**				
Q1 (Lowest)	2,364	-	-	20.70 (20.49 - 20.92)
Q2	2,363	-	-	21.40 (21.18 - 21.63)
Q3	2,363	-	-	21.30 (21.09 - 21.52)
Q4	2,363	-	-	21.29 (21.07 - 21.51)
Q5 (Highest)	2,362	-	-	21.44 (21.22 - 21.67)

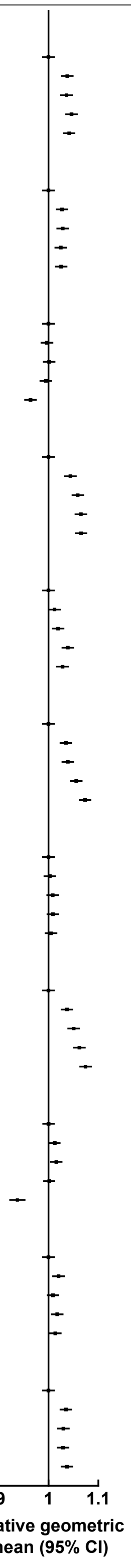

Supplementary Figure S5. Minimally adjusted models for percentage of energy from carbohydrates sources, fibre, alcohol, and energy intake in association with geometric mean concentrations of IGF-I in the WebQ 24-hour dietary assessment subsample (N=11,815)

All models are adjusted for sex and age at recruitment.

Supplementary Methods and Materials

Percentage of energy from carbohydrate sources, fibre quintiles, alcohol categories, and energy intake quintiles calculated from a minimum of four (maximum five) averaged 24-hour web-based dietary assessments with one assessment completed at recruitment.

Grams calculated as mean per day within each quintile and category.

Percentage of energy calculated by mean percentage of energy per day in each quintile.

Abbreviations: CI, confidence intervals; g, grams; IGF-I, insulin-like growth factor-I; N, number of participants; Q, quintile.

WebQ subsample (N=11,815)	Absolute change in nmol/L (95% CI)	Percentage change (95% CI)
Total protein	0.44 (0.34 to 0.53)	2.01% (1.58% to 2.43%)
Protein from plants	0.70 (0.50 to 0.89)	3.29% (2.39% to 4.19%)
Protein from animals	0.22 (0.14 to 0.31)	1.02% (0.63% to 1.41%)
Protein from dairy	0.83 (0.62 to 1.03)	3.88% (2.92% to 4.84%)
Protein from milk	1.33 (1.03 to 1.63)	6.15% (4.76% to 7.55%)
Protein from yogurt	1.41 (0.88 to 1.95)	6.79% (4.32% to 9.27%)
Protein from cheese	-0.04 (-0.37 to 0.29)	-0.12% (-1.64% to 1.41%)
Protein from animal products (not dairy)	0.09 (0.01 to 0.18)	0.39% (0.00% to 0.79%)
Total carboyhydrates	0.12 (0.08 to 0.16)	0.54% (0.37% to 0.71%)
Fibre (per 5 g/day)	0.48 (0.38 to 0.57)	2.25% (1.81% to 2.69%)
Total fats	-0.06 (-0.11 to -0.02)	-0.32% (-0.54% to -0.10%)

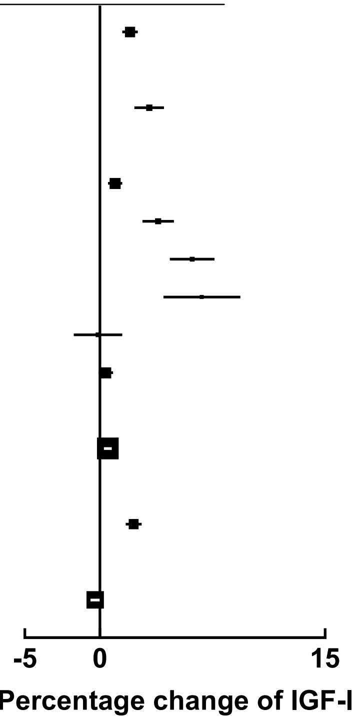

Supplementary Figure S6. Minimally adjusted models for 2.5% incremental intake of energy from macronutrients and 5 grams per day fibre intake in association with the absolute and percentage change concentration of IGF-I

All models are adjusted for sex and age at recruitment.

Macronutrients calculated from WebQ subsample with a minimum of four (maximum of five) averaged 24-hour web-based dietary assessments with one completed at recruitment.

Macronutrients are modelled as a 2.5% energy increase whereas fibre is modelled as a 5 gram per day increase.

Abbreviations: CI, confidence intervals; g/day, grams per day; IGF-I, insulin-like growth factor-I

Per 2.5% energy increase	Female (N=6,686) Absolute change in nmol/L (95% CI)	Female Percentage change (95% CI)	Male (N=5,129) Absolute change in nmol/L (95% CI)	Male Percentage change (95% CI)	c^2_1	p-value
Total protein	0.63 (0.50 to 0.75)	3.09 (2.50 to 3.68)	0.52 (0.36 to 0.67)	2.30 (1.60 to 3.00)	0.00.	0.977
Protein from plant sources	0.45 (0.19 to 0.71)	2.23 (1.00 to 3.47)	0.72 (0.42 to 1.03)	3.17 (1.80 to 4.55)	1.79	0.181
Protein from animal sources	0.41 (0.29 to 0.52)	2.00 (1.47 to 2.52)	0.26 (0.12 to 0.40)	1.18 (0.55 to 1.80)	0.44	0.508
Protein from dairy sources	0.64 (0.37 to 0.91)	3.01 (1.75 to 4.28)	0.91 (0.58 to 1.24)	4.33 (2.84 to 5.82)	3.68	0.055
Protein from milk	1.34 (0.95 to 1.73)	6.23 (4.41 to 8.05)	1.03 (0.53 to 1.53)	4.96 (2.73 to 7.18)	0.00.	0.991
Protein from yogurt	1.15 (0.51 to 1.78)	5.74 (2.75 to 8.72)	2.08 (1.09 to 3.06)	9.32 (4.93 to 13.71)	3.37	0.066
Protein from cheese	-0.54 (-0.98 to -0.10)	-2.59 (-4.65 to -0.52)	0.45 (-0.04 to 0.94)	2.22 (-0.02 to 4.41)	8.00.	0.005
Protein from animal products (not dairy)	0.30 (0.19 to 0.42)	1.51 (0.98 to 2.04)	0.10 (-0.04 to 0.24)	0.42 (-0.20 to 1.05)	2.35	0.125
Total carbohydrate	0.05 (0.00 to 0.11)	0.24 (0.00 to 0.50)	0.11 (0.05 to 0.17)	0.48 (0.19 to 0.76)	2.62	0.106
Total sugars	0.02 (-0.04 to 0.07)	0.08 (-0.19 to 0.35)	0.03 (-0.04 to 0.09)	0.07 (-0.24 to 0.38)	0.05	0.825
Free sugars	-0.20 (-0.28 to -0.12)	-0.99 (-1.37 to -0.62)	-0.18 (-0.26 to -0.10)	-0.88 (-1.25 to -0.51)	0.11	0.738
Non-free sugars	0.16 (0.09 to 0.22)	0.76 (0.45 to 1.07)	0.24 (0.15 to 0.32)	1.07 (0.68 to 1.46)	4.15	0.042
Total starch	0.06 (-0.01 to 0.13)	0.28 (-0.04 to 0.60)	0.13 (0.05 to 0.21)	0.63 (0.28 to 0.99)	3.23	0.072
Starch from wholegrains	0.23 (0.14 to 0.32)	1.15 (0.72 to 1.59)	0.25 (0.16 to 0.35)	1.19 (0.76 to 1.61)	0.2	0.654
Starch from refined grains	0.10 (0.02 to 0.17)	0.42 (0.08 to 0.77)	-0.01 (-0.09 to 0.07)	0.02 (-0.34 to 0.37)	2.07	0.15
Fibre (per 5g/day)	0.37 (0.21 to 0.52)	1.83 (1.10 to 2.56)	0.55 (0.38 to 0.72)	2.41 (1.67 to 3.15)	0.09	0.767
Total fat	-0.15 (-0.22 to -0.09)	-0.76 (-1.06 to -0.46)	-0.04 (-0.11 to 0.04)	-0.08 (-0.43 to 0.26)	3.75	0.053
Fat from plant sources	-0.10 (-0.18 to -0.02)	-0.52 (-0.91 to -0.13)	0.03 (-0.07 to 0.12)	0.15 (-0.28 to 0.58)	2.39	0.122
Fat from animal sources	-0.10 (-0.17 to -0.03)	-0.49 (-0.81 to -0.17)	-0.05 (-0.13 to 0.02)	-0.18 (-0.52 to 0.16)	0.86	0.355
Fat from dairy sources	-0.13 (-0.23 to -0.03)	-0.68 (-1.15 to -0.21)	-0.01 (-0.12 to 0.11)	0.06 (-0.44 to 0.56)	2.38	0.123
Fat from milk	0.51 (0.20 to 0.81)	2.24 (0.79 to 3.69)	0.01 (-0.34 to 0.36)	-0.01 (-1.57 to 1.55)	3.82	0.051
Fat from yogurt	0.36 (-0.22 to 0.94)	1.71 (-1.03 to 4.45)	0.94 (0.12 to 1.76)	4.83 (1.18 to 8.48)	1.82	0.177
Fat from cheese	-0.29 (-0.47 to -0.12)	-1.45 (-2.27 to -0.62)	0.11 (-0.08 to 0.31)	0.59 (-0.26 to 1.44)	8.72	0.003
Fat from animal products (not dairy)	-0.06 (-0.15 to 0.02)	-0.26 (-0.65 to 0.13)	-0.08 (-0.17 to 0.02)	-0.31 (-0.74 to 0.11)	0.02	0.894

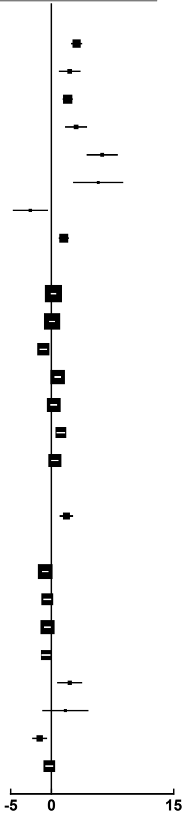

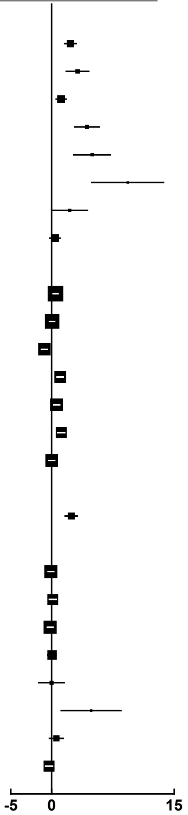

Supplementary Figure S7. Multivariable adjusted model for nutrient intake per 2.5% increase of energy from carbohydrates, fats, and proteins and 5 grams per day increase in fibre **by sex** in association with IGF-I

All models are adjusted for age at recruitment, region of recruitment, body mass index, height, physical activity, Townsend deprivation index, education, smoking, alcohol consumption, ethnicity, diabetes, energy intake and women specific covariates: hormone replacement therapy use, oral contraceptive use, and menopausal status.

χ^2 and p-values for heterogeneity represent values from likelihood ratio tests for adding an interaction term between sex and 2.5% energy increase for macronutrients or 5 g/day increase of fibre and testing for significant model fit. Bonferroni correction: p-values <0.00185 considered statistically significant.

Abbreviations: CI, confidence intervals; g/day, grams per day; IGF-I, insulin-like growth factor-I.

Per 2.5% energy increase	BMI <30 kg/m² (N=9,606) Absolute change in nmol/L (95% CI)	Percentage change (95% CI)	BMI ≥ 30 kg/m² (N=2,187) Absolute change in nmol/L (95% CI)	Percentage change (95% CI)	c_1^2	p-value
Total protein	0.58 (0.47 to 0.69)	2.32 (1.85 to 2.78)	0.45 (0.23 to 0.67)	2.43 (1.33 to 3.52)	0.56	0.456
Protein from plant sources	0.52 (0.31 to 0.74)	2.28 (1.32 to 3.24)	0.44 (-0.06 to 0.94)	2.36 (-0.24 to 4.96)	0.30.	0.583
Protein from animal sources	0.34 (0.25 to 0.44)	1.42 (1.01 to 1.84)	0.31 (0.11 to 0.51)	1.77 (0.74 to 2.81)	0.01	0.937
Protein from dairy sources	0.60 (0.37 to 0.84)	2.52 (1.49 to 3.55)	1.24 (0.75 to 1.72)	6.85 (4.35 to 9.34)	8.71	0.003
Protein from milk	1.14 (0.80 to 1.48)	4.91 (3.42 to 6.39)	1.73 (0.98 to 2.47)	9.16 (5.34 to 12.99)	2.83	0.093
Protein from yogurt	1.27 (0.68 to 1.87)	5.38 (2.76 to 8.01)	1.31 (0.06 to 2.55)	8.54 (2.09 to 14.99)	0.44	0.507
Protein from cheese	-0.33 (-0.70 to 0.04)	-1.64 (-3.27 to 0.01)	0.71 (0.03 to 1.46)	3.95 (0.06 to 7.84)	8.31	0.004
Protein from animal products (not dairy)	0.25 (0.15 to 0.34)	1.05 (0.62 to 1.47)	0.10 (-0.11 to 0.30)	0.64 (-0.41 to 1.70)	1.41	0.236
Total carbohydrate	0.08 (0.03 to 0.13)	0.35 (0.15 to 0.56)	0.05 (-0.04 to 0.14)	0.18 (-0.31 to 0.66)	3.54	0.06
Total sugars	0.02 (-0.03 to 0.07)	0.09 (-0.13 to 0.31)	0.00 (-0.09 to 0.10)	-0.03 (-0.54 to 0.48)	0.67	0.414
Free sugars	-0.18 (-0.24 to -0.11)	-0.79 (-1.08 to -0.51)	-0.21 (-0.34 to -0.08)	-1.19 (-1.85 to -0.54)	1.19	0.276
Non-free sugars	0.18 (0.12 to 0.24)	0.75 (0.50 to 1.01)	0.18 (0.06 to 0.30)	0.96 (0.36 to 1.56)	0.01	0.907
Total starch	0.09 (0.03 to 0.15)	0.43 (0.18 to 0.69)	0.08 (-0.05 to 0.20)	0.34 (-0.30 to 0.98)	2.34	0.126
Starch from wholegrains	0.21 (0.14 to 0.28)	0.95 (0.63 to 1.26)	0.32 (0.14 to 0.49)	1.74 (0.86 to 2.63)	0.22	0.639
Starch from refined grains	0.07 (0.01 to 0.13)	0.31 (0.04 to 0.58)	0.00 (-0.12 to 0.13)	0.00 (-0.60 to 0.67)	2.07	0.15
Fibre (per 5 g/day)	0.42 (0.30 to 0.55)	1.64 (1.16 to 2.12)	0.48 (0.21 to 0.74)	1.52 (0.31 to 2.73)	0.28	0.598
Total fat	-0.11 (-0.17 to -0.06)	-0.44 (-0.68 to -0.20)	-0.04 (-0.15 to 0.07)	-0.20 (-0.76 to 0.37)	0.02	0.901
Fat from plant sources	-0.02 (-0.09 to 0.04)	-0.08 (-0.38 to 0.22)	-0.12 (-0.27 to 0.04)	-0.62 (-1.41 to 0.17)	5.07	0.024
Fat from animal sources	-0.10 (-0.16 to -0.05)	-0.43 (-0.68 to -0.18)	0.02 (-0.09 to 0.14)	0.14 (-0.47 to 0.75)	2.35	0.125
Fat from dairy sources	-0.11 (-0.19 to -0.02)	-0.50 (-0.86 to -0.13)	0.03 (-0.15 to 0.21)	0.20 (-0.74 to 1.14)	2.17	0.141
Fat from milk	0.26 (0.00 to 0.51)	1.00 (-0.13 to 2.13)	0.48 (-0.10 to 1.05)	2.79 (-0.19 to 5.78)	1.37	0.242
Fat from yogurt	0.54 (0.03 to 1.05)	2.44 (0.16 to 4.71)	0.06 (-1.26 to 1.37)	2.09 (-4.74 to 8.91)	0.01	0.906
Fat from cheese	-0.18 (-0.33 to -0.04)	-0.87 (-1.51 to -0.23)	0.16 (-0.13 to 0.46)	0.90 (-0.66 to 2.46)	6.43	0.011
Fat from animal products (not dairy)	-0.08 (-0.15 to -0.01)	-0.30 (-0.61 to 0.01)	0.02 (-0.12 to 0.15)	0.08 (-0.64 to 0.79)	0.42	0.518

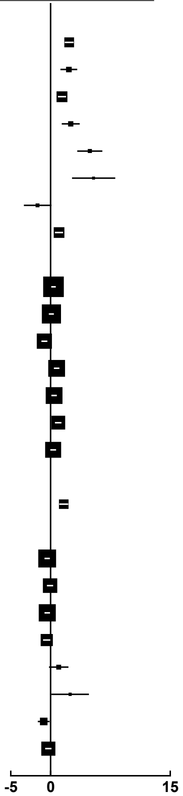

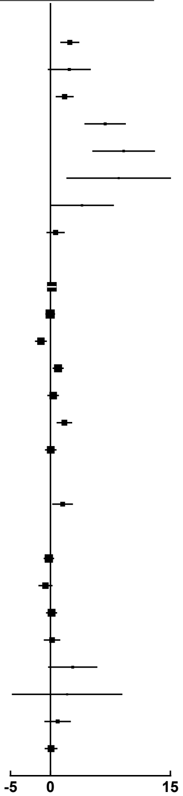

Supplementary Figure S8. Multivariable-adjusted model for nutrient intake per 2.5% increase of energy from carbohydrates, fats, and proteins and 5 grams per day increase in fibre **by body mass index (<30 and ≥30 kg/m²)** in association with IGF-I

All models are adjusted for sex, age at recruitment, region of recruitment, height, physical activity, Townsend deprivation index, education, smoking, alcohol consumption, ethnicity, diabetes, energy intake, and women specific covariates: hormone replacement therapy use, oral contraceptive use, and menopausal status. Participants with unknown BMI excluded from analyses.

χ^2 and p-values for heterogeneity represent values from likelihood ratio tests for adding an interaction term between BMI subgroups and 2.5% energy increase for macronutrients or 5g/day increase of fibre and testing for significant model fit. Bonferroni correction: p-values <0.00185 considered statistically significant.

Abbreviations: BMI, body mass index; CI, confidence intervals; g/day, grams per day; IGF-I, insulin-like growth factor-I.

WebQ subsample	N	Mean g/day	Percentage of energy	Geometric mean IGF-I concentration (nmol/L)
**Total protein**				
Q1 (Lowest)	1,182	63.6	11.72%	19.90 (19.61 - 20.19)
Q2	1,182	72.5	13.30%	20.73 (20.43 - 21.02)
Q3	1,181	75.9	14.12%	20.88 (20.58 - 21.18)
Q4	1,182	77.9	14.80%	21.13 (20.83 - 21.43)
Q5	1,181	79.8	15.44%	21.23 (20.93 - 21.53)
Q6	1,182	82.7	16.10%	21.61 (21.31 - 21.92)
Q7	1,182	83.6	16.78%	21.50 (21.20 - 21.81)
Q8	1,181	85.3	17.57%	21.33 (21.03 - 21.64)
Q9	1,182	87.2	18.67%	21.93 (21.61 - 22.24)
Q10 (Highest)	1,180	92.4	21.12%	22.13 (21.81 - 22.46)
**Protein from plants**				
Q1 (Lowest)	1,182	19.7	3.75%	20.50 (20.20 - 20.79)
Q2	1,182	23.2	4.41%	20.68 (20.39 - 20.98)
Q3	1,181	24.8	4.75%	20.96 (20.66 - 21.26)
Q4	1,182	26	5.04%	21.19 (20.89 - 21.49)
Q5	1,181	27.2	5.31%	21.32 (21.02 - 21.62)
Q6	1,182	28.1	5.58%	21.74 (21.44 - 22.05)
Q7	1,182	29.8	5.88%	21.39 (21.09 - 21.70)
Q8	1,181	31.1	6.25%	21.56 (21.25 - 21.87)
Q9	1,182	33.3	6.79%	21.27 (20.97 - 21.57)
Q10 (Highest)	1,180	39.7	8.27%	21.71 (21.40 - 22.03)
**Protein from animal**				
Q1 (Lowest)	1,182	27.2	5.18%	20.31 (20.01 - 20.60)
Q2	1,182	40.4	7.52%	20.91 (20.61 - 21.21)
Q3	1,181	45.9	8.55%	20.81 (20.52 - 21.11)
Q4	1,182	49.3	9.30%	21.15 (20.85 - 21.45)
Q5	1,181	52.2	9.96%	21.39 (21.09 - 21.70)
Q6	1,182	54.4	10.62%	21.64 (21.33 - 21.95)
Q7	1,182	56.7	11.34%	21.43 (21.13 - 21.74)
Q8	1,181	59.7	12.13%	21.10 (20.80 - 21.40)
Q9	1,182	62.8	13.25%	21.80 (21.49 - 22.11)
Q10 (Highest)	1,180	69.6	15.77%	21.79 (21.48 - 22.12)
**Protein from dairy**				
Q1 (Lowest)	1,182	3.9	0.75%	20.36 (20.07 - 20.66)
Q2	1,182	7.7	1.49%	20.94 (20.65 - 21.25)
Q3	1,181	9.8	1.88%	21.04 (20.74 - 21.34)
Q4	1,182	11.1	2.20%	21.07 (20.77 - 21.37)
Q5	1,181	12.6	2.48%	21.02 (20.72 - 21.32)
Q6	1,182	13.9	2.76%	21.71 (21.41 - 22.02)
Q7	1,182	15.1	3.06%	21.36 (21.06 - 21.67)
Q8	1,181	16.6	3.41%	21.59 (21.29 - 21.90)
Q9	1,182	18.9	3.89%	21.55 (21.25 - 21.86)
Q10 (Highest)	1,180	22.8	5.00%	21.66 (21.35 - 21.98)
**Protein from milk**				
Q1 (Lowest)	1,182	0.3	0.05%	20.78 (20.49 - 21.08)
Q2	1,182	2.6	0.50%	20.48 (20.19 - 20.78)
Q3	1,181	4.3	0.84%	20.70 (20.41 - 21.00)
Q4	1,182	5.6	1.08%	20.96 (20.67 - 21.26)
Q5	1,181	6.6	1.28%	21.54 (21.24 - 21.85)
Q6	1,182	7.5	1.48%	21.32 (21.02 - 21.62)
Q7	1,182	8.4	1.68%	21.39 (21.09 - 21.70)
Q8	1,181	9.2	1.90%	21.50 (21.20 - 21.81)
Q9	1,182	10.3	2.19%	21.63 (21.32 - 21.94)
Q10 (Highest)	1,180	12.9	2.94%	22.02 (21.70 - 22.34)
**Total Fat**				
Q1 (Lowest)	1,182	46.7	22.32%	21.42 (21.11 - 21.73)
Q2	1,182	56.7	26.13%	21.54 (21.23 - 21.85)
Q3	1,181	62.5	28.07%	21.64 (21.34 - 21.96)
Q4	1,182	66.4	29.56%	21.14 (20.84 - 21.44)
Q5	1,181	70.6	30.89%	21.31 (21.01 - 21.62)
Q6	1,182	74.8	32.15%	21.24 (20.94 - 21.55)
Q7	1,182	78.4	33.52%	20.96 (20.66 - 21.26)
Q8	1,181	83.2	35.03%	21.17 (20.87 - 21.47)
Q9	1,182	88.6	36.91%	21.09 (20.79 - 21.39)
Q10 (Highest)	1,180	98.3	40.75%	20.79 (20.49 - 21.09)

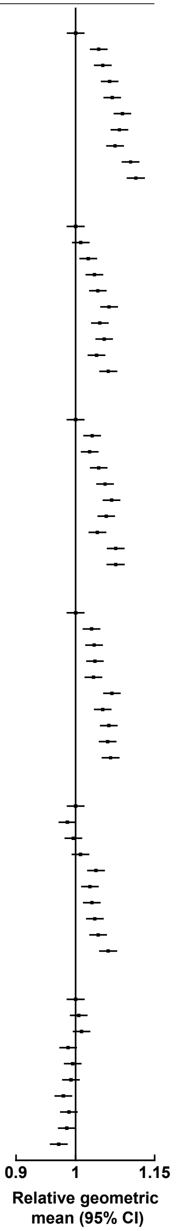

WebQ subsample	N	Mean g/day	Percentage of energy	Geometric mean IGF-I concentration (nmol/L)
**Total carbohydrate**				
Q1 (Lowest)	1,182	189.1	36.79%	20.75 (20.44 - 21.06)
Q2	1,182	221.8	42.58%	20.89 (20.59 - 21.20)
Q3	1,181	234.1	45.11%	21.35 (21.04 - 21.66)
Q4	1,182	246.6	47.11%	21.42 (21.11 - 21.73)
Q5	1,181	249.8	48.80%	21.29 (20.99 - 21.59)
Q6	1,182	257	50.37%	21.24 (20.94 - 21.55)
Q7	1,182	266.5	51.97%	21.22 (20.92 - 21.52)
Q8	1,181	272.7	53.81%	21.51 (21.21 - 21.82)
Q9	1,182	279.9	56.16%	21.20 (20.90 - 21.51)
Q10 (Highest)	1,180	288.3	60.76%	21.43 (21.11 - 21.75)
**Non-free sugar**				
Q1 (Lowest)	1,182	30.4	5.82%	20.06 (19.77 - 20.36)
Q2	1,182	42	8.09%	20.95 (20.65 - 21.26)
Q3	1,181	49.4	9.57%	21.17 (20.87 - 21.47)
Q4	1,182	55.2	10.82%	21.18 (20.88 - 21.49)
Q5	1,181	60.5	12.01%	21.27 (20.97 - 21.58)
Q6	1,182	65	13.19%	21.61 (21.30 - 21.92)
Q7	1,182	72	14.49%	21.47 (21.16 - 21.78)
Q8	1,181	78.5	16.10%	21.52 (21.21 - 21.83)
Q9	1,182	88.3	18.34%	21.39 (21.09 - 21.70)
Q10 (Highest)	1,180	107.2	23.59%	21.70 (21.39 - 22.02)
**Starch from wholegrain**				
Q1 (Lowest)	1,182	0.6	0.10%	20.56 (20.26 - 20.86)
Q2	1,182	7.3	1.40%	20.78 (20.48 - 21.08)
Q3	1,181	13.2	2.50%	21.06 (20.76 - 21.36)
Q4	1,182	18.3	3.60%	21.26 (20.96 - 21.57)
Q5	1,181	22.9	4.50%	21.28 (20.98 - 21.58)
Q6	1,182	27.4	5.40%	21.05 (20.76 - 21.35)
Q7	1,182	32.2	6.40%	21.43 (21.13 - 21.74)
Q8	1,181	38	7.70%	21.39 (21.09 - 21.70)
Q9	1,182	44.7	9.30%	21.57 (21.26 - 21.88)
Q10 (Highest)	1,180	62.8	13.20%	21.93 (21.62 - 22.25)
**Fibre**				
Q1 (Lowest)	1,182	10.1	-	20.14 (19.84 - 20.45)
Q2	1,182	12.9	-	20.77 (20.47 - 21.07)
Q3	1,181	14.4	-	21.15 (20.85 - 21.45)
Q4	1,182	15.7	-	20.97 (20.67 - 21.27)
Q5	1,181	17	-	21.29 (20.99 - 21.60)
Q6	1,182	18.2	-	21.33 (21.03 - 21.64)
Q7	1,182	19.5	-	21.49 (21.19 - 21.80)
Q8	1,181	21.1	-	21.57 (21.27 - 21.89)
Q9	1,182	23.2	-	21.77 (21.45 - 22.09)
Q10 (Highest)	1,180	28.4	-	21.85 (21.52 - 22.18)
**Alcohol**				
Q1 (Lowest)	1,425	0	0.00%	21.82 (21.48 - 22.16)
Q2	939	0.6	0.20%	21.64 (21.26 - 22.03)
Q3	1,181	2.9	0.90%	21.36 (21.04 - 21.69)
Q4	1,182	6.8	2.10%	21.40 (21.09 - 21.72)
Q5	1,181	10.6	3.40%	21.38 (21.07 - 21.69)
Q6	1,182	15.2	4.80%	21.24 (20.94 - 21.55)
Q7	1,182	20.2	6.50%	21.24 (20.93 - 21.55)
Q8	1,181	26.3	8.60%	21.19 (20.88 - 21.50)
Q9	1,182	35	11.40%	20.75 (20.43 - 21.07)
Q10 (Highest)	1,180	53.9	18.20%	20.27 (19.94 - 20.60)

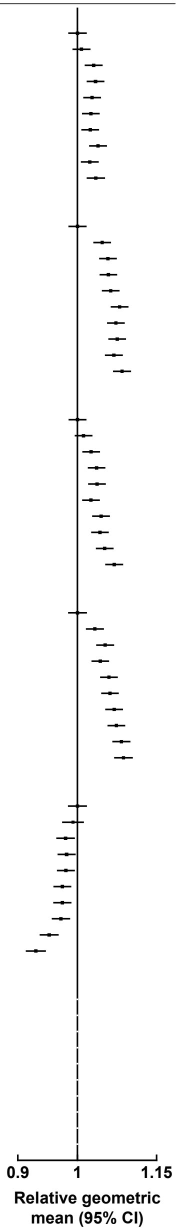

Supplementary Figure S9. Multivariable-adjusted model for percentage of energy **in deciles** from several macronutrients and fibre from the WebQ 24-hour dietary assessment subsample in association with the geometric mean concentration of IGF-I (N=11,815)

All models are adjusted for sex, age at recruitment, region of recruitment, body mass index, height, physical activity, Townsend deprivation index, education, smoking, alcohol consumption (except when alcohol was the exposure), ethnicity, diabetes, energy intake, and women specific covariates: hormone replacement therapy use, oral contraceptive use, and menopausal status.

Quantiles of percentage of energy from carbohydrate sources, percentage of energy from fat sources, percentage of protein sources, and percentage of energy from alcohol, and fibre quantiles calculated from a minimum of four (maximum of five) averaged 24-hour web-based dietary assessments with one assessment completed at recruitment.

Mean grams per day calculated as average gram intake per day within each category.

Percentage of energy calculated by mean percentage of energy per day in each quintile.

Abbreviations: g/day, grams per day; IGF-I, insulin-like growth factor-I; N, number of participants; Q, quantile.

Controlling for fibre intake

WebQ subsample	N	Mean g/day	Percentage of energy	Geometric mean IGF-I concentration (nmol/L)
**Total protein**				
Q1 (Lowest)	2,364	68.1	12.51%	20.33 (20.13 - 20.54)
Q2	2,363	76.9	14.46%	21.02 (20.81 - 21.24)
Q3	2,363	81.3	15.77%	21.42 (21.20 - 21.63)
Q4	2,363	84.5	17.17%	21.40 (21.19 - 21.62)
Q5 (Highest)	2,362	89.8	19.89%	22.00 (21.77 - 22.23)
**Protein from plant sources**				
Q1 (Lowest)	2,364	21.5	4.08%	20.92 (20.67 - 21.17)
Q2	2,363	25.4	4.89%	21.20 (20.98 - 21.42)
Q3	2,363	27.7	5.44%	21.51 (21.30 - 21.73)
Q4	2,363	30.4	6.07%	21.33 (21.11 - 21.56)
Q5 (Highest)	2,362	36.5	7.53%	21.18 (20.93 - 21.43)
**Protein from animal sources**				
Q1 (Lowest)	2,364	33.8	6.35%	20.41 (20.20 - 20.63)
Q2	2,363	47.6	8.92%	20.95 (20.74 - 21.17)
Q3	2,363	53.3	10.29%	21.53 (21.31 - 21.75)
Q4	2,363	58.2	11.73%	21.34 (21.13 - 21.56)
Q5 (Highest)	2,362	66.2	14.51%	21.94 (21.71 - 22.17)
**Protein from dairy sources**				
Q1 (Lowest)	2,364	5.8	1.12%	20.66 (20.45 - 20.87)
Q2	2,363	10.5	2.04%	21.08 (20.87 - 21.30)
Q3	2,363	13.3	2.62%	21.36 (21.15 - 21.57)
Q4	2,363	15.9	3.23%	21.46 (21.25 - 21.68)
Q5 (Highest)	2,362	20.9	4.44%	21.59 (21.37 - 21.81)
**Protein from milk**				
Q1 (Lowest)	2,364	1.4	0.28%	20.59 (20.38 - 20.80)
Q2	2,363	5	0.96%	20.85 (20.64 - 21.06)
Q3	2,363	7.1	1.38%	21.45 (21.24 - 21.67)
Q4	2,363	8.8	1.79%	21.44 (21.22 - 21.65)
Q5 (Highest)	2,362	11.6	2.57%	21.83 (21.61 - 22.06)
**Protein from yogurt**				
Q1 (Lowest)	4,247	0	0.00%	20.97 (20.81 - 21.13)
Q2	715	1	0.18%	21.01 (20.62 - 21.41)
Q3	2,128	1.5	0.30%	21.26 (21.03 - 21.48)
Q4	2,363	2.8	0.57%	21.43 (21.22 - 21.65)
Q5 (Highest)	2,362	5.2	1.16%	21.53 (21.32 - 21.75)
**Protein from cheese**				
Q1 (Lowest)	2,364	0.2	0.04%	21.20 (20.99 - 21.42)
Q2	2,363	2.1	0.40%	21.19 (20.97 - 21.40)
Q3	2,363	3.8	0.74%	21.28 (21.06 - 21.49)
Q4	2,363	6	1.16%	21.27 (21.06 - 21.48)
Q5 (Highest)	2,362	10.3	2.06%	21.20 (20.99 - 21.42)
**Protein from animal products (not dairy)**				
Q1 (Lowest)	2,364	19.6	3.65%	20.63 (20.42 - 20.85)
Q2	2,363	33.5	6.28%	21.15 (20.94 - 21.36)
Q3	2,363	39.8	7.65%	21.35 (21.14 - 21.57)
Q4	2,363	45.3	9.04%	21.42 (21.21 - 21.64)
Q5 (Highest)	2,362	54.5	11.73%	21.59 (21.37 - 21.82)

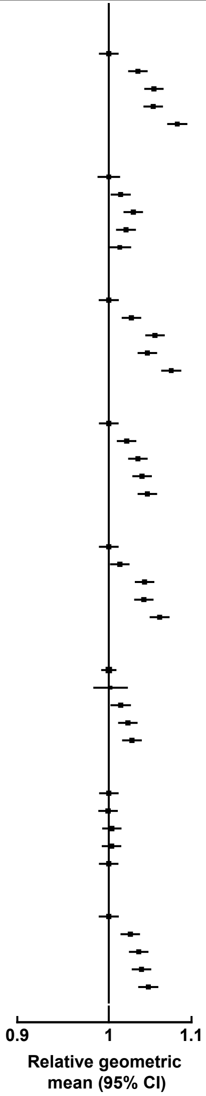

Supplementary Figure S10. Multivariable adjusted model for percentage of energy from protein **adjusting for fibre intake** in association with geometric mean concentration of IGF-I

All models are adjusted for sex, age at recruitment, region of recruitment, body mass index, height, physical activity, Townsend deprivation index, education, smoking, alcohol consumption, ethnicity, diabetes, energy intake, and women specific covariates: hormone replacement therapy use, oral contraceptive use, and menopausal status and fibre (quintiles).

Quintiles of percentage of energy from protein sources calculated from a minimum of four averaged 24-hour web-based diet assessments with one assessment completed at recruitment.

Mean grams per day calculated as average gram intake per day within each category.

Percentage of energy calculated by mean percentage of energy per day in each quintile.

Abbreviations: g/day, grams per day; IGF-I, insulin-like growth factor-I; N, number of participants; Q, quintile.

				Controlling for milk & yogurt protein intake	Controlling for fibre intake	Controlling for milk & yogurt protein and fibre intake
WebQ subsample	N	Mean g/day	Percentage of energy	Geometric mean IGF-I concentration (nmol/L)	Geometric mean IGF-I concentration (nmol/L)	Geometric mean IGF-I concentration (nmol/L)
**Total fat**						
Q1 (Lowest)	2,364	51.7	24.23%	21.38 (21.16 - 21.60)	21.34 (21.12 - 21.56)	21.24 (21.02 - 21.47)
Q2	2,363	64.5	28.81%	21.33 (21.12 - 21.55)	21.33 (21.12 - 21.55)	21.27 (21.06 - 21.49)
Q3	2,363	72.7	31.52%	21.25 (21.04 - 21.46)	21.27 (21.06 - 21.49)	21.24 (21.03 - 21.46)
Q4	2,363	80.8	34.27%	21.12 (20.91 - 21.33)	21.11 (20.90 - 21.33)	21.17 (20.96 - 21.38)
Q5 (Highest)	2,362	93.5	38.83%	21.06 (20.85 - 21.28)	21.08 (20.86 - 21.30)	21.21 (20.99 - 21.43)
**Fat from plant sources**						
Q1 (Lowest)	2,364	19.2	8.81%	20.97 (20.75 - 21.18)	21.19 (20.97 - 21.41)	21.08 (20.86 - 21.30)
Q2	2,363	26.2	11.62%	21.27 (21.06 - 21.48)	21.37 (21.15 - 21.58)	21.30 (21.09 - 21.52)
Q3	2,363	31.2	13.54%	21.47 (21.25 - 21.68)	21.50 (21.28 - 21.71)	21.47 (21.26 - 21.69)
Q4	2,363	36.3	15.64%	21.27 (21.06 - 21.48)	21.20 (20.99 - 21.42)	21.24 (21.02 - 21.45)
Q5 (Highest)	2,362	47.3	19.96%	21.17 (20.95 - 21.39)	20.89 (20.68 - 21.11)	21.05 (20.83 - 21.27)
**Fat from animal sources**						
Q1 (Lowest)	2,364	24.3	10.98%	21.41 (21.19 - 21.63)	21.14 (20.92 - 21.37)	21.17 (20.95 - 21.40)
Q2	2,363	34.1	14.95%	21.43 (21.22 - 21.65)	21.37 (21.16 - 21.59)	21.35 (21.13 - 21.57)
Q3	2,363	40.2	17.40%	21.15 (20.94 - 21.37)	21.19 (20.97 - 21.40)	21.15 (20.94 - 21.36)
Q4	2,363	46.7	20.02%	21.09 (20.88 - 21.30)	21.19 (20.97 - 21.40)	21.18 (20.96 - 21.39)
Q5 (Highest)	2,362	57.7	24.74%	21.06 (20.85 - 21.28)	21.25 (21.03 - 21.48)	21.29 (21.07 - 21.52)
**Fat from dairy sources**						
Q1 (Lowest)	2,364	3.6	1.61%	21.39 (21.17 - 21.61)	21.11 (20.90 - 21.33)	21.29 (21.08 - 21.52)
Q2	2,363	7.6	3.41%	21.31 (21.09 - 21.52)	21.27 (21.05 - 21.48)	21.27 (21.06 - 21.48)
Q3	2,363	11	4.88%	21.27 (21.06 - 21.49)	21.34 (21.13 - 21.56)	21.29 (21.08 - 21.50)
Q4	2,363	15.3	6.69%	21.18 (20.97 - 21.40)	21.29 (21.08 - 21.51)	21.22 (21.01 - 21.43)
Q5 (Highest)	2,362	24.7	10.69%	20.99 (20.77 - 21.20)	21.13 (20.91 - 21.34)	21.07 (20.85 - 21.28)
**Fat from milk**						
Q1 (Lowest)	2,364	0.3	0.13%		20.99 (20.78 - 21.21)	
Q2	2,363	1.3	0.56%		21.03 (20.82 - 21.24)	
Q3	2,363	2.7	1.17%		21.20 (20.99 - 21.42)	
Q4	2,363	3.9	1.74%		21.26 (21.05 - 21.48)	
Q5 (Highest)	2,362	6.1	2.90%		21.66 (21.44 - 21.88)	
**Fat from yogurt**						
Q1 (Lowest)	4,247	0	0.00%		20.97 (20.81 - 21.13)	
Q2	715	0.3	0.12%		20.91 (20.52 - 21.30)	
Q3	2,128	0.5	0.23%		21.35 (21.12 - 21.58)	
Q4	2,363	1.1	0.49%		21.52 (21.30 - 21.73)	
Q5 (Highest)	2,362	2.6	1.21%		21.39 (21.18 - 21.61)	
**Fat from cheese**						
Q1 (Lowest)	2,364	0.2	0.08%	21.26 (21.04 - 21.47)	21.23 (21.02 - 21.45)	21.24 (21.02 - 21.45)
Q2	2,363	2.3	0.98%	21.22 (21.01 - 21.43)	21.25 (21.03 - 21.46)	21.21 (21.00 - 21.43)
Q3	2,363	4.3	1.85%	21.21 (21.00 - 21.42)	21.24 (21.03 - 21.46)	21.21 (21.00 - 21.43)
Q4	2,363	6.8	2.95%	21.31 (21.10 - 21.53)	21.31 (21.10 - 21.53)	21.32 (21.11 - 21.54)
Q5 (Highest)	2,362	11.8	5.24%	21.14 (20.93 - 21.36)	21.11 (20.90 - 21.32)	21.15 (20.94 - 21.37)
**Fat from animal products (not dairy)**						
Q1 (Lowest)	2,364	15.4	6.77%	21.19 (20.98 - 21.41)	21.02 (20.80 - 21.25)	20.95 (20.73 - 21.18)
Q2	2,363	23	9.99%	21.27 (21.05 - 21.48)	21.23 (21.02 - 21.45)	21.19 (20.98 - 21.40)
Q3	2,363	28	11.99%	21.33 (21.12 - 21.55)	21.36 (21.15 - 21.58)	21.34 (21.12 - 21.55)
Q4	2,363	33	14.11%	21.22 (21.01 - 21.44)	21.29 (21.08 - 21.51)	21.31 (21.09 - 21.52)
Q5 (Highest)	2,362	41.5	17.97%	21.12 (20.91 - 21.34)	21.23 (21.01 - 21.45)	21.36 (21.13 - 21.58)

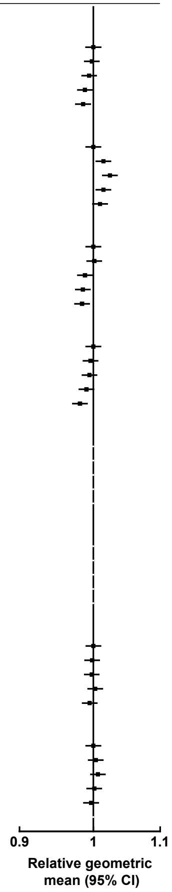

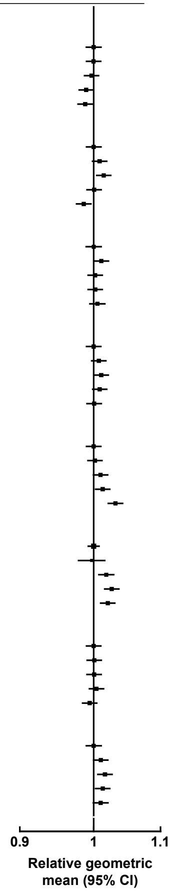

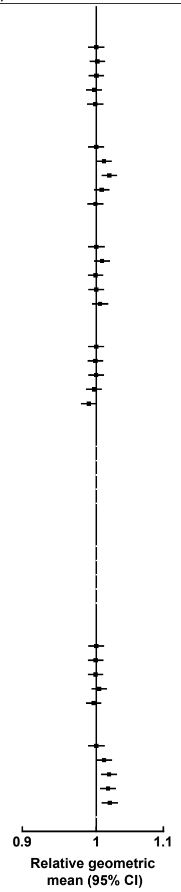

Supplementary Figure S11. Multivariable adjusted model for percentage of energy from fats **adjusting for milk and yogurt protein and fibre intake** in association with geometric mean concentration of IGF-I

All models are adjusted for sex, age at recruitment, region of recruitment, body mass index, height, physical activity, Townsend deprivation index, education, smoking, alcohol consumption, ethnicity, diabetes, energy intake, and women specific covariates: hormone replacement therapy use, oral contraceptive use, and menopausal status. The first model is adjusting for protein from milk and protein from yogurt (quintiles of

percentage of energy from protein), the middle model is further adjusted for fibre intake (quintiles), and the last model adjusted for fibre intake (quintiles) and milk and yogurt protein intake (quintiles of percentage of energy from protein).

Quintiles of percentage of energy from fat sources calculated from a minimum of four (maximum of five) averaged 24-hour web-based dietary assessments with one assessment completed at recruitment.

Mean grams per day calculated as average gram intake per day within each category.

Percentage of energy calculated by mean percentage of energy per day in each quintile.

Abbreviations: g/day, grams per day; IGF-I, insulin-like growth factor-I; N, number of participants; Q, quintile.

WebQ subsample	N	Mean g/day	Percentage of energy	Controlling for milk & yogurt protein intake: Geometric mean IGF-I concentration (nmol/L)	Controlling for fibre intake: Geometric mean IGF-I concentration (nmol/L)	Controlling for milk & yogurt protein and fibre intake: Geometric mean IGF-I concentration (nmol/L)
**Total carbohydrate**						
Q1 (Lowest)	2,364	205.4	39.68%	20.96 (20.73 - 21.18)	21.06 (20.83 - 21.30)	21.21 (20.97 - 21.44)
Q2	2,363	240.3	46.11%	21.43 (21.22 - 21.65)	21.47 (21.26 - 21.70)	21.53 (21.31 - 21.75)
Q3	2,363	253.4	49.59%	21.24 (21.03 - 21.46)	21.26 (21.05 - 21.47)	21.24 (21.02 - 21.45)
Q4	2,363	269.6	52.89%	21.29 (21.08 - 21.51)	21.26 (21.04 - 21.48)	21.19 (20.97 - 21.41)
Q5 (Highest)	2,362	284.1	58.45%	21.21 (20.99 - 21.44)	21.08 (20.86 - 21.32)	20.98 (20.75 - 21.21)
**Total sugar**						
Q1 (Lowest)	2,364	84.2	16.54%	21.27 (21.05 - 21.49)	21.24 (21.02 - 21.47)	21.42 (21.19 - 21.65)
Q2	2,363	108.7	21.14%	21.43 (21.21 - 21.64)	21.42 (21.20 - 21.63)	21.49 (21.27 - 21.70)
Q3	2,363	123.5	24.09%	21.29 (21.08 - 21.50)	21.32 (21.11 - 21.54)	21.28 (21.07 - 21.50)
Q4	2,363	137.2	27.19%	21.11 (20.90 - 21.33)	21.12 (20.91 - 21.33)	21.05 (20.84 - 21.26)
Q5 (Highest)	2,362	161.4	32.97%	21.04 (20.83 - 21.27)	21.04 (20.82 - 21.26)	20.91 (20.69 - 21.13)
**Total free sugars**						
Q1 (Lowest)	2,364	28.1	5.63%	21.56 (21.35 - 21.78)	21.48 (21.25 - 21.70)	21.45 (21.23 - 21.67)
Q2	2,363	44.6	8.67%	21.31 (21.10 - 21.52)	21.27 (21.06 - 21.49)	21.25 (21.04 - 21.46)
Q3	2,363	56.2	10.78%	21.36 (21.14 - 21.57)	21.35 (21.14 - 21.57)	21.35 (21.13 - 21.56)
Q4	2,363	68.9	13.14%	21.24 (21.03 - 21.46)	21.28 (21.07 - 21.50)	21.28 (21.07 - 21.50)
Q5 (Highest)	2,362	93.1	17.70%	20.68 (20.47 - 20.89)	20.76 (20.55 - 20.98)	20.81 (20.60 - 21.03)
**Total non-free sugar**						
Q1 (Lowest)	2,364	36.2	6.96%	20.74 (20.51 - 20.96)	20.78 (20.54 - 21.02)	21.12 (20.86 - 21.37)
Q2	2,363	52.3	10.19%	21.23 (21.02 - 21.45)	21.26 (21.04 - 21.48)	21.35 (21.13 - 21.57)
Q3	2,363	62.8	12.60%	21.41 (21.20 - 21.63)	21.43 (21.22 - 21.65)	21.41 (21.19 - 21.62)
Q4	2,363	75.2	15.29%	21.39 (21.17 - 21.61)	21.38 (21.16 - 21.60)	21.24 (21.02 - 21.46)
Q5 (Highest)	2,362	97.7	20.96%	21.38 (21.15 - 21.61)	21.29 (21.05 - 21.53)	21.03 (20.78 - 21.28)
**Total starch**						
Q1 (Lowest)	2,364	92.1	17.89%	20.99 (20.77 - 21.21)	21.09 (20.87 - 21.31)	21.09 (20.87 - 21.31)
Q2	2,363	117.5	22.46%	20.99 (20.78 - 21.21)	21.07 (20.86 - 21.28)	21.04 (20.83 - 21.25)
Q3	2,363	129.9	24.96%	21.15 (20.94 - 21.36)	21.17 (20.95 - 21.38)	21.14 (20.93 - 21.35)
Q4	2,363	140.9	27.44%	21.57 (21.35 - 21.78)	21.52 (21.31 - 21.74)	21.53 (21.32 - 21.75)
Q5 (Highest)	2,362	156.3	31.80%	21.45 (21.23 - 21.67)	21.29 (21.07 - 21.52)	21.34 (21.12 - 21.56)
**Starch from wholegrain**						
Q1 (Lowest)	2,364	3.9	0.75%	20.80 (20.59 - 21.01)	20.87 (20.65 - 21.10)	21.02 (20.79 - 21.25)
Q2	2,363	15.7	3.05%	21.17 (20.96 - 21.38)	21.24 (21.02 - 21.46)	21.26 (21.04 - 21.47)
Q3	2,363	25.2	4.93%	21.15 (20.94 - 21.36)	21.15 (20.94 - 21.37)	21.14 (20.93 - 21.35)
Q4	2,363	35.1	7.04%	21.36 (21.15 - 21.57)	21.33 (21.11 - 21.54)	21.27 (21.05 - 21.49)
Q5 (Highest)	2,362	53.7	11.27%	21.67 (21.45 - 21.90)	21.55 (21.32 - 21.79)	21.46 (21.23 - 21.69)
**Starch from refined grain**						
Q1 (Lowest)	2,364	5.4	1.02%	21.05 (20.84 - 21.26)	20.94 (20.72 - 21.15)	20.92 (20.70 - 21.13)
Q2	2,363	19.3	3.67%	21.13 (20.91 - 21.34)	21.08 (20.87 - 21.30)	21.07 (20.86 - 21.29)
Q3	2,363	31.2	5.99%	21.24 (21.03 - 21.46)	21.24 (21.02 - 21.45)	21.24 (21.02 - 21.45)
Q4	2,363	44.5	8.62%	21.32 (21.11 - 21.54)	21.38 (21.16 - 21.59)	21.39 (21.17 - 21.60)
Q5 (Highest)	2,362	67.6	13.78%	21.40 (21.19 - 21.62)	21.51 (21.29 - 21.73)	21.53 (21.31 - 21.76)
**Fibre**						
Q1 (Lowest)	2,364	11.5	-	20.50 (20.28 - 20.73)		
Q2	2,363	15.1	-	21.07 (20.86 - 21.29)		
Q3	2,363	17.6	-	21.28 (21.07 - 21.49)		
Q4	2,363	20.3	-	21.50 (21.28 - 21.72)		
Q5 (Highest)	2,362	25.8	-	21.81 (21.57 - 22.05)		
**Alcohol**						
<1 grams/day	2,551	0.03	-	21.40 (21.19 - 21.62)	21.37 (21.15 - 21.58)	21.29 (21.08 - 21.51)
1-9 grams/day	2,852	5.39	-	21.37 (21.17 - 21.57)	21.37 (21.17 - 21.57)	21.30 (21.11 - 21.50)
10-19 grams/day	2,440	14.69	-	21.43 (21.22 - 21.64)	21.45 (21.24 - 21.67)	21.41 (21.20 - 21.62)
20-39.9 grams/day	2,626	28.49	-	21.19 (20.99 - 21.40)	21.19 (20.99 - 21.40)	21.26 (21.06 - 21.47)
40+ grams/day	1,346	56.6	-	20.33 (20.05 - 20.62)	20.34 (20.05 - 20.64)	20.56 (20.26 - 20.86)
**Energy**						
Q1 (Lowest)	2,364	-	-	20.94 (20.73 - 21.16)	21.34 (21.11 - 21.57)	21.25 (21.02 - 21.49)
Q2	2,363	-	-	21.31 (21.09 - 21.52)	21.48 (21.26 - 21.70)	21.42 (21.20 - 21.63)
Q3	2,363	-	-	21.17 (20.96 - 21.39)	21.15 (20.94 - 21.36)	21.15 (20.93 - 21.36)
Q4	2,363	-	-	21.34 (21.13 - 21.56)	21.19 (20.98 - 21.41)	21.22 (21.01 - 21.44)
Q5 (Highest)	2,362	-	-	21.38 (21.16 - 21.60)	20.99 (20.76 - 21.21)	21.11 (20.88 - 21.34)
**Energy excluding alcohol**						
Q1 (Lowest)	2,364	-	-	20.76 (20.55 - 20.98)	21.14 (20.91 - 21.37)	21.07 (20.84 - 21.31)
Q2	2,363	-	-	21.39 (21.18 - 21.61)	21.53 (21.32 - 21.76)	21.50 (21.28 - 21.72)
Q3	2,363	-	-	21.24 (21.03 - 21.46)	21.23 (21.02 - 21.45)	21.22 (21.01 - 21.43)
Q4	2,363	-	-	21.30 (21.08 - 21.51)	21.14 (20.92 - 21.36)	21.16 (20.95 - 21.38)
Q5 (Highest)	2,362	-	-	21.45 (21.24 - 21.67)	21.10 (20.87 - 21.33)	21.19 (20.96 - 21.42)

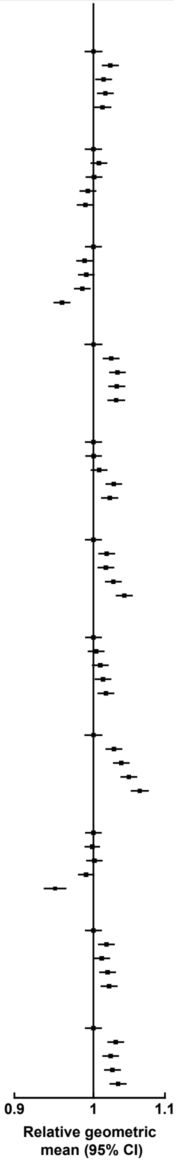

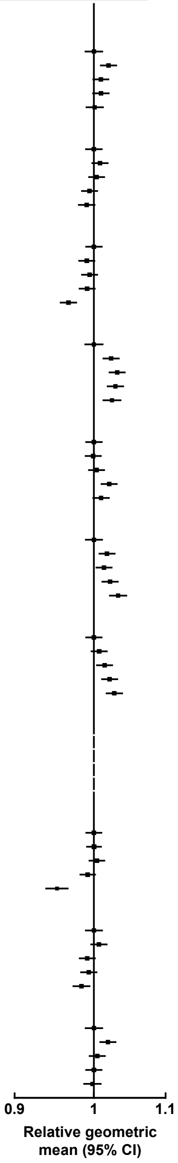

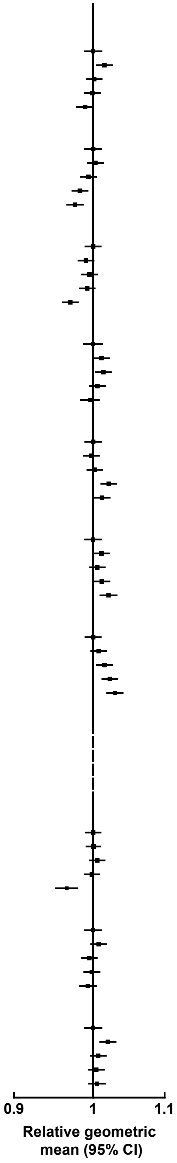

Supplementary Figure S12. Multivariable adjusted model for percentage of energy from carbohydrates, and alcohol, and energy further **adjusting for milk and yogurt protein intake and fibre intake** in association with geometric mean concentration of IGF-I

All models are adjusted for sex, age at recruitment, region of recruitment, body mass index, height, physical activity, Townsend deprivation index, education, smoking, alcohol consumption (except when alcohol was the exposure), ethnicity, diabetes, energy intake (except when energy intake was the exposure of interest), and women specific covariates: hormone replacement therapy use, oral contraceptive use, and menopausal status. The first model is adjusting for protein from milk and protein from yogurt (quintiles of percentage of energy from protein), the middle model is further adjusted for fibre intake (quintiles), and the last model adjusted for fibre intake (quintiles) and milk protein and yogurt intake (quintiles of percentage of energy from protein).

Quintiles of percentage of energy from carbohydrate sources, fibre quintiles, alcohol categories, and energy intake quintiles calculated from a minimum of four averaged 24-hour web-based diet assessments with one assessment completed at recruitment.

Mean grams per day calculated as average gram intake per day within each category.

Percentage of energy calculated by mean percentage of energy in each quintile.

Abbreviations: g/day, grams per day; IGF-I, insulin-like growth factor-I; N, number of participants; Q, quintile.
